# Defining and detecting poor outcome in heterogeneous surgical cohorts: a proof-of-concept composite distribution- and MCID-based method for study-arm-level evidence synthesis

**DOI:** 10.1016/j.mex.2026.104232

**Published:** 2026-10-23

**Authors:** Kieran Dey, Brandon Karamveer Singh Sangha, Alan Norrish

**Affiliations:** a Trauma and Orthopaedic Department, Nottingham University Hospitals NHS Trust, Nottingham, United Kingdom; b Academic Unit of Injury, Recovery and Inflammation Sciences, School of Medicine, NIHR Nottingham Biomedical Research Centre, University of Nottingham, Queen’s Medical Centre, Lenton Road, Nottingham, NG7 2UH, United Kingdom

**Keywords:** Composite outcome definition, Minimal clinically important difference, Distribution-based threshold, Synthesis without meta-analysis, Study-arm-level analysis, Sensitivity analysis, Systematic review methodology

## Abstract

Surgical outcome studies are often too heterogeneous to pool and rarely report individual patient data, so which patients do badly, and why, cannot be answered directly. Aggregate data can show which study arms did badly and what those arms had in common, but only if poor outcome is defined in advance and applied the same way to every study. We describe a reproducible method for doing this at study-arm level when meta-analysis is inappropriate. It was developed in a single systematic review (17 study arms from 13 studies) and is presented as a proof of concept that needs testing in other datasets. In the worked example, three of four classified arms held under every alternative return-time threshold, but classification was sensitive to the scoring rule, the minimal clinically important differences (MCIDs) used and missing return times.

• A composite poor-outcome definition combines distribution-based thresholds (the mean plus one standard deviation across arms) with published MCIDs, borrowed only under explicit criteria.
• Each classification is traceable to the events behind it and is tested against alternative cut-offs, thresholds and leave-one-study-out derivations.
• A Synthesis Without Meta-analysis (SWiM) moderator analysis reports each moderator’s direction, size and confidence using explicit rating rules, at study-arm level only.

## Graphical abstract

The graphical abstract is provided as a separate file.

**Figure:**
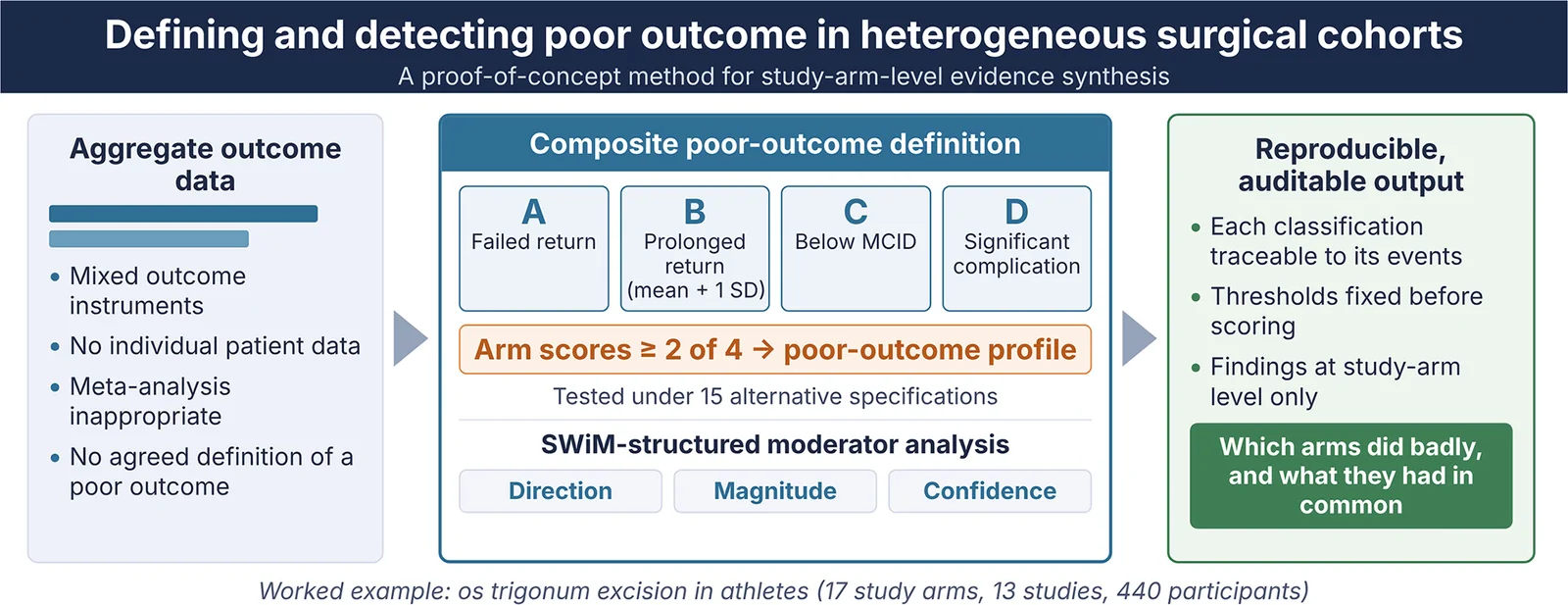

## Specifications table

**Table:** 

**Subject area**	Medicine and Dentistry
**More specific subject area**	Evidence synthesis and research methodology; outcome measurement in orthopaedic and sports-medicine surgery
**Name of your method**	Composite distribution- and MCID-based definition of poor outcome, applied at study-arm level, with Synthesis Without Meta-analysis (SWiM)-structured moderator (factor) analysis
**Name and reference of original method**	The method integrates and customises three established methods: the Synthesis Without Meta-analysis (SWiM) reporting guideline (Campbell M, McKenzie JE, Sowden A, et al. BMJ 2020;368:l6890); distribution-based and anchor-based minimal clinically important difference (MCID) thresholds (Tausendfreund J, et al. Clin Orthop Surg 2026;18:337–342; Sutton RM, et al. Foot Ankle Int 2019;40:687–693; Çelik D, et al. J Clin Epidemiol 2019;113:44–57); all applied within the PRISMA 2020 framework (Page MJ, et al. BMJ 2021;372:n71).
**Resource availability**	All analyses were performed in R v4.4.1 [1]; pooled estimates used the meta package [2], and the classification and its sensitivity analyses use base R only (Supplementary File S1; run in R v4.3.3). Screening used Rayyan [3]; risk of bias used ROBINS-I [4] and RoB 2 [5]; certainty used Grading of Recommendations, Assessment, Development and Evaluation (GRADE) [6]. The complete worked dataset is provided as Supplementary Table S1: the data-extraction table (Part A) and the moderator-coding table (Part B) for all 17 study arms, allowing the method to be reproduced end to end. No proprietary software or reagents are required.

## Background

Comparative surgical literature routinely presents two obstacles at once. First, reporting is heterogeneous: outcome instruments differ between studies, the same endpoint (return to sport, say) is defined in incompatible ways, follow-up varies widely, and event rates for the hard endpoints are low. Second, individual patient data are rarely available, so risk factors cannot be modelled at the patient level and only study-arm aggregates can be analysed. When both hold, conventional pairwise meta-analysis is usually inappropriate, which matches Cochrane guidance that a structured synthesis without meta-analysis is preferable when heterogeneity is severe [7]; PRISMA 2020 asks for the methods of such a synthesis to be reported [8].

A third problem is less obvious: the primary studies rarely agree on what counts as a poor result. Reviews therefore fall back on pooling means, and a mean functional score of 94 or a mean return time of nine weeks describes the average patient but says nothing about the minority who never regain their prior level or who suffer a serious complication. If no threshold for a poor result is set in advance, any cut-off chosen afterwards is open to manipulation, and the question that actually drives practice, which arms did badly and what they had in common, cannot be answered the same way twice.

We developed the method for exactly this situation. It defines a poor outcome before any analysis is done, combining a distribution-based threshold (calculated from the spread of the included data rather than borrowed from another population) with anchor-based minimal clinically important difference (MCID) thresholds [[9], [10], [11]] wherever these have been published for the instrument in use, and then applies that definition the same way to every study arm. A pre-specified set of candidate moderators is then examined against the resulting classification under the Synthesis Without Meta-analysis (SWiM) framework [12], with direction, magnitude and confidence reported for each. None of the parts is new; the contribution is in how they are combined, drawing on the SWiM reporting guideline, distribution- and anchor-based MCID methodology, and the PRISMA 2020 framework. We built the method during a systematic review of open versus endoscopic excision of a symptomatic os trigonum in athletes. Nothing in its steps is tied to that question, but it has so far been applied to that single dataset of 17 study arms, so we present it as a proof of concept: a fully specified and reproducible procedure whose classification we have tested for stability, but not yet validated against patient-level outcomes or in a second clinical area. It is intended for sets of aggregate comparative outcome data in which success is reported inconsistently and a reproducible, auditable definition of poor outcome is needed.

## Method details

The method proceeds in ten steps. Steps 1–6 build and apply the composite poor-outcome definition; steps 7–8 conduct the moderator (factor) analysis; steps 9–10 add the supplementary quantitative procedures and the sensitivity analyses, including those that test the classification itself. Throughout, the worked figures shown in square brackets are those obtained when the method was applied to the companion dataset (17 study arms from 13 studies; Supplementary Table S1), and are given only to illustrate each step; they are not required inputs. R code reproducing the classification and all of its sensitivity analyses is provided as Supplementary File S1; it was drafted with Claude Opus 5.5 (Anthropic), a generative AI model, and checked by the authors, who confirmed that it reproduces every reported value.

**Step 1. Define the unit of analysis and the outcome hierarchy**

Set the unit of analysis to the study arm rather than the study, so that each technique arm of a comparative study is treated separately [worked example: one randomised controlled trial (RCT) and three non-randomised comparative studies each contributed two arms (one of these compared posterior endoscopy with subtalar arthroscopy, analysed as a third technique) and nine single-arm studies one each, giving 17 arms across 13 studies]. Pre-specify an outcome hierarchy that structures both extraction and synthesis. In the worked example the hierarchy was: (1) primary: failed return to prior level (binary count); (2) co-primary: time to full return (weeks); (3) safety: any complication, neurological complications, wound complications (counts), with residual symptoms and reoperation recorded separately; (4–5) functional and patient-reported scores, extracted instrument by instrument together with their baseline values, which both the MCID criterion and any MCID normalisation require (Step 4); and (6) any domain-specific additional outcome. Fix this hierarchy before extraction.

**Step 2. Extract data against the hierarchy**

Two reviewers independently extract each arm onto a piloted, standardised table, resolving disagreements by consensus or a third reviewer. Extract study identification and design (including level of evidence), sample size, population characteristics, and the outcome hierarchy above. For outcomes reported at more than one milestone, such as return to training versus return to full participation at the prior level, record the milestone each study used, because thresholds must be derived and applied within a single milestone (Step 3). In parallel, code every candidate moderator onto a separate coding table, recording the source page or table for each coded value so that the classification can be checked against the primary studies. The worked extraction and coding tables are provided as Supplementary Table S1 (Parts A and B).

**Step 3. Derive the distribution-based thresholds**

For each continuous outcome, calculate the mean and standard deviation (SD) of the arm-level means across the included arms, using only arms that report the outcome at the same milestone, and set the distribution-based threshold for a poor result at one SD beyond the mean in the unfavourable direction. An arm scores positive only if its mean lies strictly beyond the threshold (greater than, not equal to, for a longer-is-worse outcome), so report thresholds to enough decimal places to show on which side each arm falls. Where a study reports only a median and range, estimate its mean by the sample-size-adjusted method of Wan et al. [13]. Use a single threshold across all arms as the primary analysis. Thresholds derived within subgroups defined by a candidate moderator, such as technique-specific thresholds, should be kept for sensitivity analysis: conditioning the threshold on the moderator removes the between-group difference that the moderator analysis is designed to detect, and subgroups are often too small to give a stable SD. [Worked example, time to full or prior-level return to sport, 11 arms: mean 10.68 weeks, SD 3.87 → threshold 14.55 weeks. Technique-specific thresholds, used only as a sensitivity analysis: endoscopic 11.80 weeks (7 arms); open 16.89 weeks (4 arms). The six arms reporting only an earlier or unspecified milestone, or no return time, were not assessable for this component and scored 0; scoring them 1 instead is tested in Step 10.]

The mean plus 1 SD rule was chosen because, for an approximately normal spread of arm means, it marks roughly the least favourable sixth of the observed distribution: it identifies arms that are unusual relative to comparable evidence while leaving most arms unclassified. The threshold is therefore relative rather than absolute. It identifies results that are poor compared with the other included arms, not results that fall below an externally established clinical standard. Two consequences follow: an identical arm mean can be classified differently in two syntheses that include different studies, and if every included arm performed badly, none would be flagged. Users should therefore (i) prefer an externally established clinical cut-off where one exists for the outcome and population, such as a consensus definition of delayed return or a published patient acceptable symptom state, and treat the distribution-based threshold as secondary; (ii) report the threshold together with the arms from which it was derived; (iii) report how far the threshold moves when each study in turn is omitted; and (iv) repeat the classification with alternative distribution-based and fixed thresholds (Step 10). [Worked example: we identified no externally established cut-off for delayed return after os trigonum excision, so the distribution-based threshold was primary. Omitting each study in turn moved it between 13.23 and 15.09 weeks.]

**Step 4. Set the MCID-based functional thresholds**

For functional and patient-reported scores, apply an anchor-based MCID threshold wherever a published MCID exists for the instrument. A poor functional outcome is met by an improvement smaller than the MCID where both baseline and final scores are reported or, where only a final score is reported, by a final score below a pre-specified cut-off, taken from a published grading where one exists. Record, for each score, whether the MCID was met. [Worked example: American Orthopaedic Foot and Ankle Society (AOFAS) score [14] change < 8 points [9,15] or final < 84 (the lower limit of ‘good’ in the grading used by one included study, Nikolopoulos et al.; Supplementary Table S1); visual analogue scale (VAS) pain change < 2.0 [10] or final ≥ 2.0 (pre-specified; no published grading was used); Foot and Ankle Outcome Score (FAOS) [16] final < 75 on any subscale (pre-specified; in the one arm using the FAOS, every subscale exceeded 75).]

MCID estimates depend on the population, intervention, anchor, estimation method and follow-up interval from which they were derived [17,18], so an estimate should be transferred to another population only under explicit rules, and a principled choice is needed when several published estimates exist [19]. Table 1 sets out the criteria. An estimate is eligible for borrowing only if it meets all three mandatory criteria; among eligible estimates, those meeting the most preferred criteria are favoured and the primary value is taken at the lower end of their range (a lenient choice, so that the component flags only clear failures to improve), and the highest eligible estimate is carried forward as a sensitivity bound (Step 10). Report, for each borrowed value, which preferred criteria it met. If no estimate meets the mandatory criteria, borrow nothing and use a distribution-based threshold of half the baseline SD instead [20], labelled as such. [Worked example: no MCID has been derived in posterior ankle impingement. The AOFAS estimates came from hallux valgus surgery (7.9 to 30.2 points [15]) and unstable ankle fracture (anchor-based estimates of 5.5 and 7.8 points at one year [9]; the 4.1-point lower bound its authors give is a minimal detectable change, which fails criterion 3), and the VAS estimates from hallux valgus surgery (1.8 to 5.2 points [10]). Each source shared the instrument and the foot and ankle region, and both were judged comparable surgical populations under criterion 2, but only hallux valgus surgery met preferred criterion 4 (elective surgery). The primary values (AOFAS 8, VAS 2.0) were therefore set at the lower end of the hallux valgus estimates, and the highest estimates were tested in Step 10.]

**Table 1:** Criteria for borrowing an MCID estimate from another population.

Criterion	Type	Rationale and what to report
1. Same instrument, version, scale range and scoring direction	Mandatory	MCIDs are instrument-specific; a value for a modified version or a different subscale is not transferable.
2. Same body region and a clinically comparable condition or surgical population	Mandatory	Responsiveness and the change patients regard as important differ between regions and conditions. State the source condition and why it is judged comparable.
3. Anchor-based (or receiver-operating-characteristic) estimate using a patient-rated, interpretable anchor	Mandatory	Distribution-based values describe measurement spread, not importance to patients [18]. State the anchor.
4. Comparable intervention (for example, elective surgery rather than trauma care or non-operative treatment)	Preferred	The change considered important depends on the burden and expectations of the intervention.
5. Comparable baseline severity (source baseline mean within the range of the included arms)	Preferred	MCIDs tend to scale with baseline score; a value from a less severe population understates the change needed.
6. Comparable follow-up interval	Preferred	Estimates differ between early and late follow-up; match the interval at which final scores are assessed.
7. Credible estimation: anchor–change correlation reported (ideally ≥ 0.5) and the estimate given with a measure of precision	Preferred	Follows the credibility criteria of Devji et al. [18]. Report the correlation and the interval where available.

An estimate meeting criteria 1–3 is eligible. Among eligible estimates, those meeting the most preferred criteria are favoured; the lower end of their range is used as the primary value and the highest eligible estimate as a sensitivity bound. If no estimate is eligible, half the baseline SD [20] is used and labelled as a distribution-based threshold. MCID, minimal clinically important difference.

Where a synthesis needs a single continuous functional outcome across instruments, the MCID normalisation framework of Ramadanov [21] can be added at this step: each arm’s change score is divided by the instrument’s MCID to give a common scale in ‘MCID units’, and the SD of the change is derived from the pre- and post-operative SDs and their correlation. Within this method, component C is already equivalent to a threshold of one MCID unit applied instrument by instrument, so normalisation would change the classification only if a different cut-off in MCID units were chosen. We did not adopt normalised scores as an input to the classification, for three reasons. First, normalisation inherits the uncertainty of a borrowed MCID and, once applied, hides it: in the worked example the same AOFAS improvement of 18.9 points was 3.4 MCID units against the lowest eligible estimate (5.5 points) and 0.63 units against the highest (30.2 points). Second, the SD of the change requires the pre–post correlation, which the included studies did not report, and several arms reported no baseline score. Third, as Ramadanov notes, normalisation aligns scales but not constructs, and the instrument used most often in the worked example, the AOFAS, is partly clinician-administered. Normalised values for every arm with baseline data are given in Supplementary Table S5 Part E for users who wish to pool them; we recommend reporting them under at least two MCID anchors, the primary value and the highest eligible estimate.

**Step 5. Specify the composite components and the scoring rule**

Collapse the hierarchy into a small number of binary components, each scoring 0 or 1 per arm. In the worked example four components were used: (A) failed return to prior level, where any failure in the arm scores 1; (B) prolonged return, scored against the common distribution-based threshold from Step 3; (C) sub-MCID functional improvement, from Step 4; and (D) clinically significant complication, meaning any nerve complication, complex regional pain syndrome, wound complication requiring active management, or reoperation. Pre-specify the classification rule before scoring: in the worked example, an arm meeting ≥ 2 of the 4 components was classified as a poor-outcome profile. Components that score any event, such as A and D here, are sensitive to arm size, because large arms are more likely to record at least one event; Step 10 describes a rate-based alternative, and the dependence should be checked (Method validation).

The ≥ 2 rule is a pre-specified convention rather than an empirically derived cut-off, and no external standard exists against which an arm-level cut-off could be calibrated. Its rationale is structural. With four unweighted components, two is the lowest cut-off at which no single component can classify an arm on its own, so an isolated event, such as one transient neurapraxia in a large series, cannot by itself label an arm as poor. A stricter rule (≥ 3) demands agreement across domains that are often reported incompletely, so it tends to classify on completeness of reporting rather than on outcome. Components are unweighted because no arm-level evidence was available to justify weights. Users should report the full distribution of scores and the classification under the neighbouring cut-offs (≥ 1 and ≥ 3) as a sensitivity analysis (Step 10). [Worked example: component B was assessable in 11 of 17 arms and component C was met in none, so an arm without a reported return time could not reach a score of 3. With ≥ 1, 13 of 17 arms were classified and the definition no longer discriminated; with ≥ 3, one arm was.]

**Step 6. Score every arm and build the classification table**

Score each arm against components A–D, sum to a 0–4 score, and apply the pre-specified rule. Present the result as a per-arm table giving each component score, the total, and a short rationale citing the specific events behind each positive component, so that any reader can check a classification against the source data. [Worked example: scores were 0 in 4 arms, 1 in 9, 2 in 3 and 3 in 1. Four of the 17 arms reached the threshold, two endoscopic and two open. Component D was positive in 12 arms and component C in none, so every classified arm met at least two of components A, B and D, and no arm was classified on a single event.]

**Step 7. Pre-specify and code the candidate moderators**

List the candidate moderators a priori and code each for every arm. Treat binary contrasts (e.g. technique) as such; code continuous or proportional moderators both continuously and against a pre-specified binary threshold; and, where a moderator cannot be separated from another variable in the primary studies (a structural confound), record it but flag it for narrative rather than statistical treatment. For each moderator, also record whether a within-study contrast exists, that is, two arms of one study that differ on the moderator or a study reporting outcomes by level of the moderator, because such contrasts are protected from between-study confounding in a way that comparisons between arms are not. [Worked example: eight moderators were coded. Ossicle size, reported in one study, and concomitant pathology were presented in a single row, and technique was rated separately for return to sport and for complications, giving eight factor rows. Surgeon experience was inseparable from technique or era in two of the studies that reported it and was described narratively for those; a within-study learning-curve analysis in a third allowed it to be rated (Step 8).]

**Interpreting study-arm associations.** Every association produced by Steps 7 and 8 is an association between characteristics of study arms and the outcome profiles of study arms. Such associations can appear, disappear or reverse at patient level (ecological bias) [22,23]. An arm with a high proportion of dancers and a poor-outcome profile does not show that dancers do worse: the dancers in that arm may not be the patients who did badly, and the proportion of dancers in an arm travels with its technique, era, surgeon and definition of return to sport. Arm-level findings should therefore be reported as descriptions of the evidence base (‘arms in which X was more common more often had a poor-outcome profile’), not as patient-level prognostic factors or causal effects, and should not be used to counsel individual patients. Within-study contrasts should be reported separately and given priority, because they compare patients drawn from the same population, surgeon and protocol; even so, they remain observational unless the factor was randomised. A patient-level prognostic factor can be established only from individual patient data or from studies that report outcomes stratified by the factor.

**Step 8. SWiM-structured moderator analysis**

Analyse each moderator following the SWiM reporting guideline [12], whose nine items should be addressed explicitly in a checklist (Supplementary File S2 for the worked example). For every factor, tabulate the available data (the number of contributing arms and of within-study contrasts), the direction of effect, the magnitude of any difference where quantifiable, and a confidence rating for that direction. Assign confidence with the decision rules in Table 2 rather than by overall judgement. The rules adapt the GRADE approach [6]. A factor is first rated ‘not determinable’ if any exclusion condition applies; otherwise the starting level is set by the type of comparison available (randomised within-study, non-randomised within-study, or between arms only) and is lowered one level for each of inconsistency, imprecision and risk of bias or structural confounding, each defined by an explicit criterion. Two reviewers should apply the rules independently and report their agreement. [Worked example: risk of bias and certainty of evidence were taken from Supplementary Tables S2 and S3, and two reviewers applied the rules independently. Applied to the eight factor rows, the rules reproduced the original rating in six (low confidence for technique and return to sport, very low for technique and complications, very low for dancer status and symptom duration, and not determinable for flexor hallucis longus pathology and rehabilitation protocol, originally recorded as ‘insufficient data’ and ‘not testable’). In the combined row for ossicle size and concomitant pathology, originally rated very low, they gave very low for concomitant pathology but ‘not determinable’ for ossicle size, which was collinear with technique in the only study that measured it. For surgeon experience they gave ‘very low’, from one within-study learning-curve analysis that found no difference in clinical outcome, where the original analysis had recorded ‘not testable across arms’ (Supplementary Table S5 Part F).]

**Table 2:** Decision rules for rating confidence in the direction of a moderator’s effect.

Stage	Rule
1. Not determinable (applied first)	Rate ‘not determinable’ and report no direction if any of the following holds: the factor is reported in fewer than two arms; it does not vary across arms; it is fully collinear with another factor in every study reporting it; or it is measured in non-comparable ways across studies and no within-study contrast exists.
2. Starting level	High: the factor was randomised within a trial (rate as for the GRADE assessment of that comparison). Low: at least one non-randomised within-study contrast. Very low: comparisons between arms only.
3a. Inconsistency (−1)	Fewer than 75% of contributing within-study contrasts share the same direction of effect or, where only between-arm comparisons exist, the direction differs between the outcomes analysed (for example, return to sport and complications).
3b. Imprecision (−1 or −2)	−1 if the 95% confidence interval of the primary estimate includes no difference, or if fewer than about 400 participants (continuous outcomes) or 300 events (binary outcomes) contribute; −2 if the interval is very wide and includes both appreciable benefit and appreciable harm.
3c. Risk of bias or structural confounding (−1)	Most of the contributing information comes from studies at serious or critical risk of bias, or every contributing contrast carries an identified structural confound (for example, surgeon, era or patient selection collinear with the factor).
4. Final rating	High, moderate, low or very low (not below very low), or not determinable. Report the stage-by-stage reasons for each factor.

The 75% consistency criterion and the information-size thresholds are pre-specified conventions, the latter following GRADE guidance on imprecision [24]. The rules are applied to the worked example in Supplementary Table S5 Part F.

**Step 9. Supplementary quantitative procedures**

Where a subset of studies provides directly comparative data, compute per-study effect estimates as the primary quantitative output: odds ratios (OR) with 95% confidence intervals for binary outcomes, using Fisher’s exact test as the primary test (with the Yates-corrected chi-squared reported alongside) because expected cell counts are typically low. Report these per study rather than pooled, since differences in case mix, technique and surgeon preclude meaningful pooling. As a clearly labelled supplement, pooled estimates may be added: a random-effects mean difference for continuous outcomes (restricted maximum likelihood estimate of τ² with the Hartung–Knapp adjustment and a 95% prediction interval based on the t distribution with k − 1 degrees of freedom) and a fixed-effect Mantel–Haenszel (MH) pooled OR for binary outcomes, excluding double-zero-event studies in line with Cochrane guidance [7], always reported with heterogeneity statistics (Cochran’s Q, I², τ²). Present pooled estimates alongside, never as a substitute for, the per-study findings, and interpret them cautiously when heterogeneity is high. All calculations in the worked example were performed in R v4.4.1: base stats functions for Fisher’s exact and Yates-corrected tests, and the meta package for pooled MH ORs and random-effects mean differences. [Worked example: per-study and pooled estimates are given in Supplementary Table S4.]

**Step 10. Sensitivity analyses**

Pre-specify two kinds of sensitivity analysis. The first tests the moderator findings: remove the most likely sources of distortion and repeat the primary factor analyses within each restricted set, checking that the direction and approximate magnitude of effect are preserved [worked example: excluding dancer-predominant arms, and restricting to arms that excluded concomitant pathology]. The second tests the classification itself, varying one element of the definition at a time: (i) the classification rule (≥ 1 and ≥ 3 components); (ii) the distribution-based threshold (mean plus 0.5, 1.5 and 2 SD; a sample-size-weighted mean and SD; the 75th and 90th percentiles of arm means; fixed cut-offs not derived from the data; subgroup-specific thresholds; and an alternative milestone); (iii) leave-one-study-out derivation of the threshold, scoring every arm, including those of the omitted study, against the threshold derived without it; (iv) rate-based versions of any-event components, positive when the arm’s event rate exceeds the mean plus 1 SD of arm rates (in the worked example the rate-based version of component D used each arm’s any-complication rate, so minor complications also counted); (v) the MCID component at the highest eligible MCID estimates (Step 4); and (vi) missing values scored as positive rather than as zero, a worst case for arms that did not report a component. For each specification, report the number of arms classified, the arms whose classification changed and Cohen’s κ against the primary classification; for each arm, report the number of specifications in which it was classified. Supplementary File S1 contains code for all of these.

## Method validation

We evaluated the method on the companion dataset (17 arms, 13 studies, 440 participants, counting ankles for the one study that reported only ankles) in six ways. The first four address the poor-outcome classification itself, the fifth tests the moderator findings, and the sixth checks the extracted data against an independent synthesis. None is a validation against a patient-level reference standard, which does not exist for this question, and the results support the method as a proof of concept rather than as a validated instrument.

**Traceability.** Each of the four arms classified as a poor-outcome profile could be traced through the per-arm scoring table to the specific events behind it, and every one met at least two separate components rather than one.

**Stability across specifications.** Table 3 shows the classification under the primary specification and the 15 alternative specifications described in Step 10; the leave-one-study-out derivation is reported separately below. Under every alternative threshold for component B, three to five arms were classified, three arms (Nikolopoulos et al., López Valerio et al. and Heyer and Rose) were classified every time, and agreement with the primary classification was high (κ 0.55 to 0.85). The arms that changed were the open arm of Ballal et al. (mean return 14.9 weeks) and Sugimoto et al. (13.6 weeks), both within a week of the primary threshold of 14.55 weeks, and, with technique-specific thresholds only, Smyth et al. (11.9 weeks against an endoscopic threshold of 11.80). The classification was much less stable to the other elements of the definition: the neighbouring cut-offs classified 13 arms (≥ 1) and 1 arm (≥ 3), rate-based scoring of components A and D classified 2, and the higher MCID estimates classified 10 (κ 0.35). Scoring the six arms without a full or prior-level return time as prolonged classified 7 arms (κ 0.61), adding both arms of Ahn et al. and Walsh et al., each of which already had a positive component D. Across all 16 specifications, one arm was classified every time, four arms never, and the other twelve between 1 and 14 times (Supplementary Table S5 Part B).

**Table 3:** Sensitivity of the poor-outcome classification in the worked example (17 study arms).

Specification	Threshold	Arms classified (endoscopic / open / subtalar)	Arms changed	κ
Primary: ≥ 2 of 4; B at mean + 1 SD (common)	14.55 wk	4 (2 / 2 / 0)	–	–
Rule: ≥ 1 of 4	14.55 wk	13 (7 / 5 / 1)	9	0.17
Rule: ≥ 3 of 4	14.55 wk	1 (0 / 1 / 0)	3	0.34
B: mean + 0.5 SD	12.61 wk	5 (3 / 2 / 0)	1	0.85
B: mean + 1.5 SD	16.48 wk	3 (2 / 1 / 0)	1	0.82
B: mean + 2 SD	18.42 wk	3 (2 / 1 / 0)	1	0.82
B: sample-size-weighted mean + 1 SD	15.61 wk	3 (2 / 1 / 0)	1	0.82
B: 75th percentile of arm means	12.75 wk	5 (3 / 2 / 0)	1	0.85
B: 90th percentile of arm means	14.90 wk	3 (2 / 1 / 0)	1	0.82
B: fixed cut-off, 12 weeks	12.00 wk	5 (3 / 2 / 0)	1	0.85
B: fixed cut-off, 16 weeks	16.00 wk	3 (2 / 1 / 0)	1	0.82
B: technique-specific mean + 1 SD	11.80 (E); 16.89 (O) wk	5 (4 / 1 / 0)	3	0.55
B: best available milestone, 16 arms	13.30 wk	5 (3 / 2 / 0)	1	0.85
Missing return time scored as prolonged (B = 1)	14.55 wk	7 (3 / 3 / 1)	3	0.61
A and D rate-based (rate > mean + 1 SD of arm rates)	A 3.6%; D 12.8%	2 (0 / 2 / 0)	2	0.60
C at the higher MCID estimates	AOFAS 30.2; VAS 5.2	10 (5 / 4 / 1)	6	0.35

Arms changed, number of arms whose classification differs from the primary; κ, Cohen’s kappa against the primary classification. The fixed cut-offs are illustrative and not externally validated. Rate-based component D uses each arm’s any-complication rate. The arms classified under each specification, the leave-one-study-out analysis and the code are given in Supplementary Table S5 and Supplementary File S1. AOFAS, American Orthopaedic Foot and Ankle Society score; E, endoscopic; MCID, minimal clinically important difference; O, open; SD, standard deviation; subtalar, subtalar arthroscopy (one arm, Ahn et al.); VAS, visual analogue scale; wk, weeks.

**Dependence on the included studies.** Omitting each study in turn moved the threshold for component B between 13.23 and 15.09 weeks. Omitting a study with no full or prior-level return time cannot move the threshold, so 8 of the 13 omissions changed it; in 4 of these 8 one arm changed classification (the open arm of Ballal et al. in three, Sugimoto et al. in one). This shows the limitation of a relative threshold directly: the same mean return time of 14.9 weeks was classified as prolonged or not depending on which other studies were included.

**Dependence on arm size.** Because components A and D score any event, larger arms were more likely to score. The composite score correlated with arm size (Spearman ρ 0.66, p = 0.004), and classified arms had a median of 30 participants against 16 in unclassified arms. Rate-based scoring reduced the number of classified arms to two, both open, but did not remove the correlation (ρ 0.56, p = 0.018), because the arms with no events were mostly small and compatible with substantial event rates. Any-event components therefore partly measure arm size rather than outcome. Users should report this diagnostic, and future refinements should score event rates with their uncertainty, for example after shrinkage towards the overall rate, rather than the presence of any event.

**Robustness of the moderator findings.** Excluding the six dancer-predominant arms (more than half dancers: Shimozono et al., Morelli et al., both arms of Ballal et al., Walsh et al. and Heyer and Rose), and separately restricting to the six arms of the four studies that explicitly excluded concomitant pathology (Nikolopoulos et al., Morelli et al., Georgiannos and Bisbinas, and Ahn et al.), left the direction of the technique contrast unchanged for both return to sport and complications (Supplementary Table S5 Part G). These analyses test the moderator signal, not the classification; the clinical estimates themselves belong to the companion systematic review rather than to this method.

**Consistency of extracted data with an independent synthesis.** As a check on extraction rather than on the classification, we compared aggregate outputs with the meta-analysis of Zwiers et al. [25], which used different inclusion criteria and pooled all causes of posterior ankle impingement. Endoscopic return times agreed closely (unweighted mean 8.8 weeks across seven arms here; 8.9 weeks pooled by Zwiers et al.), open return times did not (14.0 versus 10.8 weeks), which may reflect our restriction to the full or prior-level milestone and the predominance of dancers in the open arms, and complication proportions were ordered in the same direction (open 12.2% here and 15% pooled; endoscopic 5.2% and 8%). This agreement supports the extraction. It says nothing about whether the poor-outcome classification is valid, and we therefore do not describe it as external validation.

**What validation of the classification would require.** Validating the classification needs a reference standard measured in patients: for example, individual patient data from several studies, so that the proportion of patients with a poor outcome in each arm can be compared with the arm’s classification, or prospective application in a field where such data exist. Until that has been done, and until the method has been applied to a second, independent clinical dataset, it should be regarded as a reproducible proof of concept rather than a validated instrument.

## Limitations

This is a proof of concept, developed and demonstrated in a single systematic review of 17 study arms from 13 studies. That is a small empirical base: the return-to-sport threshold rests on 11 arms, the moderator analyses on fewer, and the method’s behaviour in other clinical areas, with more arms, different outcome hierarchies or more frequent events, is untested. It should be applied to further, independent datasets before it is recommended for general use.

The method works at study-arm level. Its associations are between characteristics of study arms and their outcome profiles; they are vulnerable to ecological bias, cannot identify patient-level prognostic factors and do not support causal conclusions. This is a property of aggregate data rather than a weakness the method can correct, and it limits what any arm-level moderator analysis can claim.

The distribution-based thresholds are relative to the included evidence. They identify arms that performed poorly compared with the other included arms, not against an external clinical standard, so the same result can be classified differently in syntheses with different included studies, and a uniformly poor evidence base would generate no flags. They should be re-derived for each synthesis, reported with their leave-one-study-out range, and replaced by external cut-offs where these exist. They also need enough arms to give a stable mean and SD; in the worked example the technique-specific threshold for open surgery rested on four arms.

The ≥ 2 rule is a convention without an external reference standard, and the any-event components depend on arm size. In the worked example the threshold for component B changed the classification of one to three arms (three of the four classified arms held under every alternative), but the classification was not stable to the classification rule, rate-based scoring, the handling of missing return times or the MCID values used. The MCID thresholds were borrowed from other foot and ankle populations under explicit criteria, which make the uncertainty of a borrowed value visible but cannot remove it, and the functional component was never met. Last, the low event counts common for hard surgical endpoints limit the supplementary pooled estimates, which are best read as hypothesis-generating rather than definitive.

## Ethics statements

This method was developed and demonstrated using data extracted from previously published studies. The work did not involve human subjects, animal experiments, or data collected from social media platforms, and ethical approval was therefore not required. The authors confirm that they have read and follow the ethical requirements for publication in MethodsX.

## Supplementary material and/or additional information

The following material supports full reproduction of the method:

• Supplementary Table S1: combined data-extraction table (Part A) and moderator-coding table (Part B) for all 17 study arms, with the source page or table for each coded value. This is the worked dataset on which the method is demonstrated.
• Supplementary Table S2: risk-of-bias assessment (ROBINS-I [4] domain-level judgements for the non-randomised studies; RoB 2 [5] for the randomised trial).
• Supplementary Table S3: GRADE evidence profile for each outcome domain.
• Supplementary Table S4: quantitative synthesis: per-study and pooled estimates for time to return to sport (random-effects mean difference with prediction interval) and any complication (OR), with heterogeneity statistics, neurological complications, arm-level event rates with confidence intervals, and component scores under alternative return-time thresholds.
• Supplementary Table S5: sensitivity analyses of the poor-outcome classification: arms classified under the primary and each of the 15 alternative specifications, per-arm component scores with the events behind them and stability, leave-one-study-out thresholds, the arm-size diagnostic, MCID-normalised change scores, the confidence decision rules applied to each factor, and the moderator sensitivity analyses.
• Supplementary File S1: R code (base R) reproducing the classification and every sensitivity analysis in Table 3 and Supplementary Table S5.
• Supplementary File S2: completed SWiM reporting checklist, giving for each of the nine items the step of the method that addresses it and where it is reported for the worked example.

## Declaration of generative AI and AI-assisted technologies in the manuscript preparation process

During the preparation of this work the authors used Claude (Anthropic; Claude Opus 4.8 for the original submission and Claude Opus 5.5 during revision), a generative AI language model, to improve the readability and language of the manuscript and, during revision, to assist with drafting revised text and writing the R code for the sensitivity analyses. After using this tool, the authors reviewed, verified and edited the content and code as needed and take full responsibility for the content of the published article.

## Declaration of competing interest

The authors declare that they have no known competing financial interests or personal relationships that could have appeared to influence the work reported in this paper.

## Data Availability

Data will be made available on request.
