# Supplementary material for: Defining and detecting poor outcome in heterogeneous surgical cohorts: a proof-of-concept composite distribution- and MCID-based method for study-arm-level evidence synthesis
Source: MethodsX. 2026 Oct 23;17:104232. doi: 10.1016/j.mex.2026.104232 (PMC13742498; doi:10.1016/j.mex.2026.104232)
Supplement: Supplementary file 1 [file mmc1.zip › Supplementary Appendix SWiM.docx]

**Supplementary File S2. SWiM reporting checklist**

*Synthesis Without Meta-analysis (SWiM) reporting items (Campbell et al. [1]), mapped to the steps of the method that address each item and to where each is reported for the worked example (17 study arms from 13 studies). Step numbers refer to the Method details section of the manuscript.*

| **SWiM item** | **Description** | **Where the method addresses it** | **Where reported for the worked example** |
| --- | --- | --- | --- |
| 1a. Grouping studies for synthesis | Provide a description of, and rationale for, the groups used in the synthesis (e.g. groupings of populations, interventions, outcomes, study design). | Step 1 (study arm as the unit of analysis; arms grouped by intervention); Step 7 (pre-specified moderators, coded for every arm). | Step 1 worked example; Supplementary Table S1 Parts A and B. |
| 1b. Grouping studies for synthesis | Detail and provide rationale for any changes made subsequent to the protocol in the groups used in the synthesis. | Step 3 (common threshold primary, subgroup thresholds as sensitivity analysis); Step 10. | Step 3 worked example; Table 3. |
| 2. Describe the standardised metric and transformation methods used | Describe the standardised metric for each outcome. Explain why the metric(s) was chosen, and describe any methods used to transform the intervention effects to the standardised metric. | Step 3 (distribution-based threshold, SD imputation by the method of Wan et al. [2]); Step 4 (MCID thresholds, criteria for borrowing an MCID in Table 1, optional MCID normalisation); Step 9 (odds ratios and mean differences). | Steps 3–4 worked examples; Table 1; Supplementary Table S4; Supplementary Table S5 Part E. |
| 3. Describe the synthesis methods | Describe and justify the methods used to synthesise the effects for each outcome when it was not possible to undertake a meta-analysis of effect estimates. | Steps 5–6 (composite classification with a pre-specified rule); Step 8 (structured tabulation of direction, magnitude and confidence); Step 9 (per-study estimates primary, pooled estimates supplementary). | Steps 5–6 and 8 worked examples; Supplementary Table S4. |
| 4. Criteria used to prioritise results for summary and synthesis | Where applicable, provide the criteria used, with supporting justification, to select the particular studies, or a particular study, for the main synthesis or to draw conclusions from the synthesis (e.g. based on study design, risk of bias assessments, directness in relation to the review question). | Step 1 (pre-specified outcome hierarchy); Step 7 (within-study contrasts given priority over between-arm comparisons); Step 8 and Table 2 (starting level set by comparison type); Step 9 (randomised comparison as the primary estimate). | Step 8 worked example; Supplementary Table S5 Part F. |
| 5. Investigation of heterogeneity in reported effects | State the method(s) used to examine heterogeneity in reported effects when it was not possible to undertake a meta-analysis of effect estimates and its extensions to investigate heterogeneity. | Steps 7–8 (pre-specified moderators; interpretation at study-arm level); Step 10 (case-mix sensitivity analyses). | Method validation (“Robustness of the moderator findings”); Supplementary Table S5 Part G. |
| 6. Certainty of evidence | Describe the methods used to assess certainty of the synthesis findings. | Step 8 and Table 2 (explicit decision rules adapted from GRADE). | Supplementary Table S3 (GRADE); Supplementary Table S5 Part F. |
| 7. Data presentation methods | Describe the graphical and tabular methods used to present the effects (e.g. tables, forest plots, harvest plots). Specify key study characteristics (e.g. study design, risk of bias) used to order the studies, in the text and any tables or graphs, clearly referencing the studies included. | Step 6 (per-arm classification table with event-level rationale); Step 8 (data, direction, magnitude and confidence tabulated for each factor); Step 10 (specification table). | Table 3; Supplementary Tables S1, S2, S4 and S5. |
| 8. Reporting results | For each comparison and outcome, provide a description of the synthesised findings, and the certainty of the findings. Describe the result in language that is consistent with the question the review is designed to address, and indicate which studies contribute to the synthesis. | Step 8 (direction, magnitude and confidence with contributing arms); Step 7 (wording for study-arm associations). | Step 8 worked example; Supplementary Table S5 Parts A–G. |
| 9. Limitations of the synthesis | Report the limitations of the synthesis methods used and/or the groupings used in the synthesis, and how these affect the conclusions that can be drawn in relation to the original review question. | Limitations section; Step 3 (relative thresholds); Step 5 (cut-off and arm-size dependence); Step 7 (ecological bias). | Method validation; Limitations. |

Item descriptions are paraphrased from the SWiM reporting guideline. GRADE, Grading of Recommendations, Assessment, Development and Evaluation; MCID, minimal clinically important difference; SWiM, Synthesis Without Meta-analysis.

**References**

[1] M. Campbell, J.E. McKenzie, A. Sowden, et al., Synthesis without meta-analysis (SWiM) in systematic reviews: reporting guideline, BMJ 368 (2020) l6890. https://doi.org/10.1136/bmj.l6890.

[2] X. Wan, W. Wang, J. Liu, T. Tong, Estimating the sample mean and standard deviation from the sample size, median, range and/or interquartile range, BMC Med. Res. Methodol. 14 (2014) 135.
