# Supplementary material for: Defining and detecting poor outcome in heterogeneous surgical cohorts: a proof-of-concept composite distribution- and MCID-based method for study-arm-level evidence synthesis
Source: MethodsX. 2026 Oct 23;17:104232. doi: 10.1016/j.mex.2026.104232 (PMC13742498; doi:10.1016/j.mex.2026.104232)
Supplement: Supplementary file 1 [file mmc1.zip › Supplementary_Table_S1.docx]

**Supplementary Table S1. Combined data extraction and moderator coding table.**

**Part A. Data extraction**

*Systematic Review: Endoscopic vs Open Surgery for Os Trigonum Syndrome | Outcomes hierarchy: (1) Primary: failed return to prior sport/dance level; (2) Co-primary: time to full RTS; (3) Safety: complications; (4) Residual symptoms; (5) Functional scores; (6) FHL pathology (added outcome)*

| **Study (Year)** | **Design (LoE)** | **N** | **Population** | **Conservative Tx Required** | **Follow-up** | **(1) Failed RTS to prior level n/N (%)** | **(2) Time to full RTS (weeks, mean [range])** | **(6) FHL pathology n/N (%)** | **(3) Any complication n/N (%)** | **(3) Neurological complication n/N (%)** | **(3) Wound complication n/N (%)** | **(4) Residual symptom / reoperation n/N (%)** | **(5) Functional score (final; pre-op where reported)** |
| --- | --- | --- | --- | --- | --- | --- | --- | --- | --- | --- | --- | --- | --- |
| ***ENDOSCOPIC / POSTERIOR ARTHROSCOPIC – Single-arm case series*** | | | | | | | | | | | | | |
| Nikolopoulos et al. (2020) | Retro. case series (LoE IV) | 81 | Recreational athletes (ballet 18, dancers 15, soccer 15, basketball 13, volleyball 4, polo 5, cyclists 5, marathon 4, sprinters 2); mean age 27.4 yr (range 17–46) | Min. 3 months (rest, NSAIDs, CAM boot, PT) | Min. 2 yr (range 2–5 yr) | 5/81 (6.2%) dropped to lower activity level | 9.1 (8–12) [return to activity; level not specified] | FHL released in all cases (81/81, 100%): routine per technique; not reported as pathology-driven indication | 5/81 (6.2%) | 3/81 (3.7%): transient sural nerve neurapraxia | 1/81 (1.2%): portal drainage (fat pad atrophy) | 1 arthrofibrosis, 5 lower activity; no recurrence | AOFAS: 97.7 (85–100) at 1 yr; 99.0 (87–100) at 2 yr; pre-op 39.4. FADI: 97.9 (93–100) at 1 yr; 99.3 at 2 yr. VAS: 0.6 at 1 yr; pre-op 7.5 |
| Shimozono et al. (2025) | Retro. cohort (LoE IV) | 13 | Athletes only; ballet 7, soccer 3, lacrosse 2, tennis 1; mean age 21.1 yr (range 13–39) | Failed non-operative Rx (duration not reported) | Mean 17.1 months (range 12–34) | 0/13 (0%) | Training 3.8 (SD 1.3); full at pre-injury level 6.3 (3.1–10.6); ballet: 7.3 (4.3–10.6); non-ballet: 5.1 (3.1–8.6) | 13/13 (100%): FHL tenosynovitis debrided in all patients (inclusion required FHL tenosynovitis on MRI) | 0/13 (0%) | 0/13 (0%) | 0/13 (0%) | 1/13 local tenderness but returned to prior level; no reoperations | JSSF: 98.3 (90–100). SAFE-Q pain: 97.7; physical functioning: 98.3; sports activity: 96.8 |
| Sugimoto et al. (2021) | Retro. case series (LoE IV) | 50 OT ankles (of 72 ankles, 59 patients) | OT subgroup: level 1 (professional/national) 19, level 2 (local competitive) 16, level 3 (recreational) 15 ankles; mean age 20.7 yr (range 12–50). Whole cohort: 56 sports-related patients; mean age 21.8 yr (12–74) | Min. 3 months (rest, medication, taping) | OT subgroup mean 60.3 mo (range 24–133) | 0/49 OT ankles (whole cohort 0/70 ankles) | OT subgroup (41 ankles): training 5.0 (SD 3.0; 2–14); competition or stage 13.6 (SD 7.5; 4–36). [Whole cohort 5.3; 13.4] | 17/72 ankles (23.6%): pain also induced by FHL muscle contraction | 4/72 ankles (5.6%) (whole cohort; not attributed by cause) | 0/72 (0%) | 3/72 (4.2%): skin burns (early series, monopolar cautery); 1/72 (1.4%) wound healing/scar | 1 scar excision (reoperation), whole cohort | OT subgroup AOFAS: 79.2 ± 6.6 → 98.1 ± 4.1 [whole cohort 79.6 → 97.6] |
| Smyth et al. (2013) | Retro. case series (LoE IV) | 16 OT athletes (of 22 patients) | Athletes with OT resected (from source Table 1): basketball 4, ballet 3, soccer 3, triathlon 3, tennis 1, running 1, swimming 1; mean age 24.6 yr (range 12–44). Whole cohort: 22 patients, 18 OT, 2 Stieda, 2 soft tissue; 19 athletes | US-guided injection + PT 6–8 wk | Mean 25 mo (range 14–35) | 0/16 OT athletes (whole cohort 0/19 athletes) | OT athletes: 11.9 (7–16), n = 15, excludes 1 infection case (32 wk) [whole cohort 12 (6–16)] | 20/20 (100%) of os trigonum/Stieda process resections had FHL tenosynovitis (20/22 = 90.9% of total cohort) | 2/22 (9.1%) | 1/22 (4.5%): deep peroneal nerve dysaesthesia (resolved) | 1/22 (4.5%): infection with delayed healing requiring debridement and washout | No residual impingement symptoms reported | OT athletes: FAOS 60.9 → 89.3 (69–100); SF-12 67.6 → 87.5 [whole cohort FAOS 59 → 86 (47–100); SF-12 66 → 86] |
| López Valerio et al. (2015) | Retro. case series (LoE IV) | 20 | Soccer players only; Tegner 9–10; mean age 24.8 yr (SD 3.4; range NR) | Min. 6 wk PT (NSAIDs ± US-guided steroid injection) | Min. 2 yr (mean NR; range 2–10 yr follow-up) | 1/20 (5.0%): persistent pain, second operation, did not regain prior Tegner level | 6.7 (1.7–28.7), n = 19; mean 46.9 days (text SD 26.0 days; SD from source table 44.6 days) | FHL identified/protected in all cases; pathology rate not separately quantified | 0/20 severe complications; 1/20 (5%) persistent pain requiring 2nd op | 0/20 (0%) | 0/20 (0%) | 1/20 reoperation (persistent pain) | VAS: 0.8 (SD 1.36) at RTS (1 month post-op). Pre-op VAS: 7.5 |
| Jerosch and Fadel (2006) | Retro. case series (LoE IV) | 10 | Athletic (3 professionals: ballet dancer, swimmer, volleyball); mean age 25.9 yr (range 19–32) | Failed conservative Rx; interval onset to surgery 20 mo | Mean 25 mo (range 6–61) | 0/10 (0%): 9/10 symptom free ADL at 4 wk, all returned to sport at 8 wk | 8 (all by 8 weeks) [resumed sport; level not specified] | 4/10 (40%): FHL synovectomy performed; 3/10 (30%): tendon sheath release | 0/10 (0%) | 0/10 (0%) | 0/10 (0%) | No residual impingement; no reoperations | AOFAS: 87 (post-op). Pre-op: 43 |
| Morelli et al. (2017) | Retro. case series (LoE IV) | 12 | Professional ballet dancers; 9 female; mean age 26.3 yr (SD 9.0; range 15–47); 2 of 14 operated patients excluded by the authors for a combined osteochondral lesion of the talus | Unsatisfactory improvement after a 6-month rehabilitation protocol (rest, PT, NSAIDs, corticosteroid injection, shockwave therapy) | Mean 38.9 mo (SD 20.6; range 12–72) | 0/12 (0%): all returned to full dance activity | 8.7 (SD 0.7; 8–10) [time to start performing dance again; level not specified] | Not reported | 1/12 (8.3%): local swelling for 8 wk; no major complications | 0/12 (0%) | 0/12 (0%): no superficial or deep infection | No reoperations; no pain on forced plantarflexion at final FU; all would undergo surgery again | AOFAS: 96 (SD 5.1; 87–100). Pre-op: 67.8. VAS: 2.5 (SD 0.9). Pre-op: 7.8. Tegner: 4.3 → 9 |
| ***RANDOMISED CONTROLLED TRIAL – both arms extracted separately below*** | | | | | | | | | | | | | |
| Georgiannos and Bisbinas (2017), ENDOSCOPIC arm (Group B) | RCT (LoE II): endoscopic arm | 26 | Athletes (professional and recreational); dance/ballet 12, soccer 4, basketball 2, jumping/running 6, other 2; mean age 25.4 yr (inclusion >16 yr) | Mean 8 mo (range 6–12); rest, NSAIDs, PT, walking boot (38 pts), steroid injection (29 pts) | Mean 60 mo (SD 5) | 0/26 (0%): all returned to prior sport level | 7.12 (SD 2.25); training: 4.58 (SD 1.47) | FHL inspected and treated (debridement/shaving) as per technique in all cases; incidence not separately quantified per arm | 1/26 (3.8%) | 0/26 (0%) | 0/26 (0%) | 1/26 revision endoscopy for persistent pain (residual calcaneal process) | AOFAS: 92.38 (SD 6.62) at 5 yr. Pre-op: 65.81. VAS-FA: 93.10 at 5 yr |
| Georgiannos and Bisbinas (2017), OPEN arm (Group A) | RCT (LoE II): open arm | 26 | Athletes; dance/ballet 11, soccer 6, basketball 3, jumping/running 5, other 1; mean age 26 yr (inclusion >16 yr) | Same as Group B | Mean 60 mo (SD 5) | 0/26 (0%): all returned to prior sport level | 11.54 (SD 3.89); training: 9.58 (SD 3.98) | FHL inspected and debrided if stenosing tenosynovitis identified; incidence not separately quantified per arm | 6/26 (23.1%) [trial reports p < 0.001 vs endoscopic; Fisher's exact p = 0.099 on published counts] | 3/26 (11.5%): 2 sural neurapraxia, 1 permanent sural nerve injury | 2/26 (7.7%): wound infection (treated with antibiotics) | 1 CRPS (referred to pain clinic); all returned to sport despite complications | AOFAS: 87.50 (SD 8.06) at 5 yr. Pre-op: 66.15. VAS-FA: 91.60 at 5 yr |
| ***COMPARISON STUDIES – Endoscopic vs Open (non-RCT)*** | | | | | | | | | | | | | |
| Ballal et al. (2016), ENDOSCOPIC arm | Retro. comparative (LoE IV): endoscopic | 13 | Professional national ballet dancers; mean age 20.4 yr (range 15–30) | Min. 2 mo physio | 12 mo (clinic review at 2 wk, 3, 6 and 12 mo) | 0/13 (0%): all returned to full ballet | Full dance 9.8 (6.5–16.1) [range given as 9–20 in results text]; limited dance 2.8 (1.9–7.4) | 2/13 (15.4%) required FHL decompression: significantly lower than open arm (p=0.016) | 0/13 (0%) | 0/13 (0%) | 0/13 (0%) | No reoperations; 1 residual tenderness on examination but danced at prior level | No formal score; all asymptomatic at final FU; limited dance return: mean 2.8 wk |
| Ballal et al. (2016), OPEN arm | Retro. comparative (LoE IV): open | 22 | Professional national ballet dancers; mean age 17.6 yr (range 13–22) | Min. 2 mo physio | 12 mo (clinic review at 2 wk, 3, 6 and 12 mo) | 1/22 (4.5%): failed to return to full ballet (unrelated hip injury) | Full dance 14.9 (9–20); limited dance 6.4 (5.9–6.9) | 13/22 (59.1%) required FHL decompression: significantly higher than endoscopic arm (p=0.016) | 0/22 (0%) | 0/22 (0%) | 0/22 (0%) | No reoperations; all asymptomatic at final FU | No formal score; all asymptomatic at final FU; limited dance return: mean 6.4 wk |
| Guo et al. (2010), ENDOSCOPIC arm (Group B) | Retro. comparative (LoE IV): endoscopic | 25 | High ankle-demand sport or occupation in 16/25 (soccer 6, basketball 2, dance 2, gymnastics, diving, wrestling, weightlifting, tennis, athletics 1 each); remainder not described; mean age 24.9 yr (range 13–49) | Min. 3 months (restriction, PT, NSAIDs or steroid injection) | Mean 31.1 mo (range 12–76) | 0/25 (0%) | 6.0 (4–10); dancers 7.0, soccer 4.3 | 2/25 (8%): stenosing FHL tenosynovitis treated (resection of posteromedial tibia/talus or shaving tendon sheath) | 1/25 (4%): 1 medial heel numbness (tibial nerve sensory loss; duration not stated) | 1/25 (4%): 1 medial heel numbness (tibial nerve sensory loss; duration not stated) | 0/25 (0%) | No reoperations; 1 VAS = 3 at follow-up | AOFAS: 93.4 (75–100). VAS: 0.80. Satisfaction: 96% |
| Guo et al. (2010), OPEN arm (Group A) | Retro. comparative (LoE IV): open | 16 | High ankle-demand sport or occupation in 13/16 (dance 5, soccer 3, wushu 2, diving, wrestling, badminton 1 each); remainder not described; mean age 20.9 yr (range 13–35) | Min. 3 months | Mean 48.2 mo (range 24–86) | 0/16 (0%) | 11.9 (4–24); dancers 16.0, soccer 8.3 | 1/16 (6.3%): FHL released for stenosing tenosynovitis | 2/16 (12.5%) | 1/16 (6.3%): permanent sural nerve sensory loss | 1/16 (6.3%): 10° loss dorsiflexion (wound issue NR separately) | NR | AOFAS: 90.1 (71–100). VAS: 1.25. Satisfaction: 87.5% |
| Ahn et al. (2013), ENDOSCOPIC arm | Retro. cohort (LoE III): endoscopic; mean OT size 12.6 × 10.4 mm | 12 | Amateur athletes (incl. 1 dancer); mean age 29.9 yr (range 18–55) | Min. 3 months (no steroid injections used) | Mean 30.7 mo (range 18–55) | 0/12 (0%): all satisfied, would repeat surgery | 8.0 (SD 1.8) [time to initiate previous sport, e.g. running and jumping] | FHL inspected for tenosynovitis/tendinitis in all cases; pre-existing FHL tendinitis was an exclusion criterion for the study | 1/12 (8.3%) | 1/12 (8.3%): transient sural nerve dysaesthesia (resolved <6 wk) | 0/12 (0%) | No reoperations; dancers resumed en pointe at 10 wk | AOFAS: 89.9 (SD 6.65); pre-op 64.8. VAS: 1.2; pre-op 6.7. MFS: 88.4 |
| Ahn et al. (2013), ARTHROSCOPIC arm | Retro. cohort (LoE III): subtalar arthroscopy; mean OT size 11.1 × 8.8 mm | 16 | Amateur athletes (incl. 2 dancers); mean age 29.8 yr (range 17–48) | Min. 3 months | Mean 29.1 mo (range 18–52) | 0/16 (0%); 2/16 required conversion to endoscopy intra-op (technical failure 12.5%; not a failed return) | 7.5 (SD 1.6) [time to initiate previous sport, e.g. running and jumping] | FHL inspected for tenosynovitis/tendinitis in all cases; pre-existing FHL tendinitis was an exclusion criterion for the study | 1/16 (6.3%) | 1/16 (6.3%): transient sural nerve dysaesthesia (resolved <6 wk) | 0/16 (0%) | No reoperations; 2/16 required intraoperative conversion to endoscopy | AOFAS: 89.9 (SD 5.23); pre-op 63.8. VAS: 1.2; pre-op 6.3. MFS: 89.6 |
| ***OPEN SURGERY – Single arm and dancer-specific cohorts*** | | | | | | | | | | | | | |
| Walsh et al. (2020) | Retro. case series (LoE IV) | 44 pts / 54 ankles | Dancers only (ballet, jazz, modern, contemporary); 90.9% female; mean age 18.2 yr (range 9.9–50.2) | CAM boot 6 wk + PT; NSAIDs; steroid injection in refractory cases | Mean 33.4 mo (range 12–102.6) | 0/44 (0%): all reported return to prior dance level post-PT | Not measured; all returned to previous level after completing PT (protocol milestones: modified dance class 6 wk; sport-specific training 9–12 wk) | Posteromedial approach used specifically to enable FHL tenolysis/debridement in all cases; incidence of pre-existing FHL pathology not separately quantified | 6/44 patients (reported per patient: 3 scar discomfort, 2 sural nerve injury, 1 peroneus brevis tendinitis) | 2/44 (4.5%): temporary sural nerve injury (resolved) | 3/44 (6.8%): scar discomfort (resolved with PT) | Satisfaction 83%; 1 peroneus brevis tendinitis (compensation); no reoperations | VAS: 1.73 (SD 2.10); pre-op 5.39. FFI-R cumulative: 31.2. VR-12 Physical: 51.2 |
| Heyer and Rose (2017) | Retro. case series (LoE IV): open posteromedial | 40 ankles / 38 dancers | Dancers (ballet 90%); professional 20%, preprofessional 75%; mean age 19.2 yr (range 13–39); 97.5% female | Failed conservative Rx (mean 17.6 mo post-symptom onset); PT, cortisone (8 cases), electrical stim, immobilisation, NSAIDs | Mean 4 yr (range 6 mo – 12 yr) | 2/37 (5.4%) did not return to prior dance level (1 peroneal tendinitis, 1 scar limited pointe); 3 chose not to return | 7.9 (3–20) [initial return]; 17.7 (6–52) [pain-free dance] | 17/40 (42.5%) had FHL symptomatology preoperatively (triggering, clicking, decreased excursion); all 17/17 (100%) relieved postoperatively | 4/40 (10%) minor | 0/40 (0%): no neurovascular complications | 4/40 (10%): 2 keloid, 1 superficial infection, 1 wound dehiscence (all resolved) | No reoperations; all FHL symptoms resolved | VAS: 0.6 (0–6); pre-op 7.7. Satisfaction: 95% (38/40). 100% would recommend |

Abbreviations: ADL = activities of daily living; AOFAS = American Orthopaedic Foot & Ankle Society hindfoot score; CAM = controlled ankle motion; CRPS = complex regional pain syndrome; FADI = Foot & Ankle Disability Index; FAOS = Foot and Ankle Outcome Score; FHL = flexor hallucis longus; FFI-R = Foot Function Index–Revised; FU = follow-up; JSSF = Japanese Society for Surgery of the Foot scale; LoE = level of evidence; MFS = Maryland Foot Score; NR = not reported; NSAIDs = non-steroidal anti-inflammatory drugs; OT = os trigonum; PT = physiotherapy; RCT = randomised controlled trial; Retro. = retrospective; RTS = return to sport/dance; SAFE-Q = Self-Administered Foot Evaluation Questionnaire; SD = standard deviation; VAS = visual analogue scale (0–10 unless stated); VAS-FA = VAS Foot and Ankle (0–100); VR-12 = Veterans RAND 12-item survey.

*Note on FHL pathology column: studies vary substantially in how FHL involvement was ascertained and reported: some report concurrent FHL tenosynovitis/tendinitis as a proportion of the cohort (Sugimoto, Smyth, Heyer and Rose), some report the proportion requiring surgical FHL decompression/tenolysis as a procedural outcome (Ballal, Guo, Jerosch and Fadel), and others describe FHL inspection as routine technique without quantifying pathology incidence (Nikolopoulos, López Valerio, Georgiannos and Bisbinas). Morelli et al. did not report FHL findings. Ahn et al. excluded patients with pre-existing FHL tendinitis, which should be considered when comparing FHL-related findings across studies.*

**Part B. Moderator coding**

*Systematic Review: Os Trigonum Surgery in Athletes | Coded using the a priori moderator scheme for narrative synthesis. Eight candidate moderators: technique (open vs endoscopic), dancer/ballet cohort, FHL pathology, symptom duration, os trigonum size, concomitant pathology, surgeon experience/learning curve, rehabilitation protocol.*

| **Study (Year)** | **Open vs Endoscopic** | **Dancer/Ballet Cohort (% or Y/N)** | **FHL Pathology (% symptoms or % procedure)** | **Symptom Duration (mean/median, mo)** | **Large OT (mean size or 'large'/conversion)** | **Concomitant Pathology (excluded vs included)** | **Surgeon Experience / Learning Curve Reported** | **Rehab Protocol (early WB/ROM vs immobilisation)** |
| --- | --- | --- | --- | --- | --- | --- | --- | --- |
| ***ENDOSCOPIC / POSTERIOR ARTHROSCOPIC – Single-arm case series*** | | | | | | | | |
| **Nikolopoulos et al. (2020)** | **Endoscopic** | 33/81 (40.7%) (18 ballet + 15 other dancers) | FHL released in 81/81 (100%): routine technique, not pathology-driven | Not reported (only conservative Tx duration given: 6 wk–3 mo) | Not reported (no size data; no conversions) | EXCLUDED: PAIS due to cartilage/soft-tissue pathology, ankle instability (>10° talar tilt or >10mm displacement), active infection, restricted blood supply, combined ant.+post. arthroscopy | Not reported; no learning curve analysis | Early WB: partial WB on crutches 3 wk → full WB; CAM boot; ROM exercises started ASAP post-op; sport-specific rehab ~5–6 wk |
| **Shimozono et al. (2025)** | **Endoscopic (needle, 1.9mm)** | 7/13 (53.8%) ballet | 13/13 (100%): FHL tenosynovitis debrided in all (inclusion criterion required FHL tenosynovitis on MRI) | Not reported | Not reported | Not explicitly stated as exclusion; inclusion required OT/Stieda process + FHL tenosynovitis on imaging | Not reported; single senior surgeon (Y.S.) for all cases; no learning curve analysis | Early WB: elastic bandage 2 days; full WB per pain tolerance from day 1; formal PT at 2 wk; jogging/sport-specific training at 2 wk |
| **Sugimoto et al. (2021)** | **Endoscopic** | Dancers not isolated as a cohort; ballet dancer and gymnast cited as case examples only (% NR) | 17/72 ankles (23.6%): pain induced by FHL muscle contraction | Not reported | 'Large posterior talar process' as separate aetiological category (14/72, 19.4%); no OT-specific size or conversion data | INCLUDED soft-tissue impingement (8/72) and large talar process (14/72) alongside OT (50/72): heterogeneous aetiology in same cohort; EXCLUDED loose body, bipartite talus, <24 mo follow-up | YES: explicit learning curve analysis: 26 cases required for proficiency; 2 operators new to posterior arthroscopy; surgical time fell from ~80 to ~50 min over series | Early WB: compression bandage 24h; early ROM immediately post-op; walk without crutches from next day; jog at 2 wk |
| **Smyth et al. (2013)** | **Endoscopic** | 3/16 (18.8%) ballet among OT athletes (ages 14, 15, 15) | 20/20 (100%) of bony impingement (OT/Stieda) cases had FHL tenosynovitis (20/22 = 90.9% of total cohort) | Not reported | Not reported (qualitative: 1 'unusually large' OT requiring portal extension: no measurement) | INCLUDED osteochondral lesions, lateral ligament pathology, ganglion cyst, soft-tissue impingement alongside OT/Stieda: heterogeneous; EXCLUDED <1 yr follow-up, systemic joint disease | Not reported; 2 senior authors (J.G.K., D.S.L.); no learning curve analysis; systematic 4-quadrant technique described but not tied to experience | Early WB: orthopaedic post-op shoe; gradual WB from day 2; full WB by day 7; early ROM (ankle pumps) from day 1; sport-specific PT at 2 wk |
| **López Valerio et al. (2015)** | **Endoscopic** | 0/20 (0%): soccer players only | FHL identified/protected in all cases; pathology rate not separately quantified | Mean 8.6 mo (SD 4.3) | Not reported | INCLUDED: Tegner 9–10 soccer players only (homogeneous by design); EXCLUDED previous foot/ankle surgery, infectious or tumour disease | Not reported; single surgical team; no learning curve analysis | Early WB: full WB with crutches day 1, without crutches/cane from day 5; sport activity from week 2 (bike/swim), running week 3, return to play week 5 |
| **Jerosch and Fadel (2006)** | **Endoscopic** | 1/10 (10%) ballet dancer | 4/10 (40%) FHL synovectomy performed; 3/10 (30%) tendon sheath release | Mean 20 mo (interval onset of pain to surgery, sample size n=10) | Not reported | Not explicitly stated; appears OT-specific cohort (3 professionals: ballet, swimmer, volleyball) | Not reported; no learning curve analysis; very small series (n=10) | No cast/splint applied; partial WB allowed day 1 for 14 days, then full WB |
| **Morelli et al. (2017)** | **Endoscopic** | 12/12 (100%): professional ballet dancers | Not reported | Mean 10.5 mo (SD 2.4; range 7–14) | Not reported (no size data; no conversions) | EXCLUDED: combined osteochondral lesion of the talus (2 of 14 operated patients), previous surgery on either ankle | Not reported; no learning curve analysis | Compressive bandage, non-weight-bearing for 2 wk with active dorsiflexion from day 2; weight-bearing and PT from 2 wk; running at 4 wk; dance-specific training at 6 wk |
| ***RANDOMISED CONTROLLED TRIAL – both arms extracted separately below*** | | | | | | | | |
| **Georgiannos and Bisbinas (2017), ENDOSCOPIC arm** | **Endoscopic** | 12/26 (46.2%) dance/ballet | FHL inspected/treated per technique in all cases; incidence not separately quantified per arm | Mean 8 mo conservative Tx (range 6–12); symptom-onset-to-surgery duration not separately reported | Not reported | EXCLUDED: osteochondritis dissecans, grade 2–4 ankle osteoarthritis, Achilles tendinitis: relatively 'clean' OT cohort | Not reported; single-surgeon RCT (surgical technique held constant); no learning curve analysis | No immobilisation; full WB as pain allowed; early ROM exercises encouraged; formal PT at 2 wk |
| **Georgiannos and Bisbinas (2017), OPEN arm** | **Open** | 11/26 (42.3%) dance/ballet | FHL inspected and debrided if stenosing tenosynovitis identified; incidence not separately quantified per arm | Same as endoscopic arm (shared inclusion/conservative Tx criteria) | Not reported | Same exclusion criteria as endoscopic arm | Not reported; same surgical team; no learning curve analysis | Same post-op protocol as endoscopic arm: no immobilisation; full WB as pain allowed |
| ***COMPARISON STUDIES – Endoscopic vs Open (non-RCT)*** | | | | | | | | |
| **Ballal et al. (2016), ENDOSCOPIC arm** | **Endoscopic** | 13/13 (100%): professional national ballet dancers only | 2/13 (15.4%) required FHL decompression: significantly lower than open arm (p=0.016) | Median 6 mo (range 3–26) | Not reported | EXCLUDED: previous hindfoot surgery, amateur ballet, non-recognised school/national group: homogeneous elite dancer cohort; concurrent Broström lateral ligament reconstruction in 1/13 | Not reported; 2 different senior surgeons by technique (J.D.F.C. endoscopic, R.L.W. open): surgeon and technique are fully confounded in this study | Same post-op protocol both arms: full WB as tolerated in post-op shoe; NSAIDs 2 wk; PT from day 5; clinic review 2 wk/3/6/12 mo |
| **Ballal et al. (2016), OPEN arm** | **Open** | 22/22 (100%): professional national ballet dancers only | 13/22 (59.1%) required FHL decompression: significantly higher than endoscopic arm (p=0.016) | Median 7 mo (range 2–60) in source table; 7.5 mo in source text | Not reported | Same exclusion criteria as endoscopic arm; concurrent Broström lateral ligament reconstruction in 3/22 | Not reported; surgeon R.L.W. performed all open cases; surgeon/technique confounded as above | Same post-op protocol as endoscopic arm |
| **Guo et al. (2010), ENDOSCOPIC arm (Group B)** | **Endoscopic** | 2/25 (8%) dancing (per Table 1: 2 dancing, vs soccer 6, gymnastics 1 etc.) | 2/25 (8%) stenosing FHL tenosynovitis treated | Not reported as discrete figure for this arm (cohort spans 1994–2007, technique evolved over time) | Not reported (no size data; no conversions) | INCLUDED concomitant grade I–II anterior impingement in 14 patients (4 open, 10 endoscopic), with anterior osteophyte excision at the same sitting in 5 (split by arm not reported); otherwise OT-focused | Not reported as formal learning curve, BUT technique evolved temporally: open only pre-2002, mixed 2002–2006, endoscopic only post-2006: introduces surgeon-experience/temporal confounding | Early WB: no immobilisation either arm; protected WB as tolerated; early ROM 3 days post-op; strengthening/proprioception from 2 wk |
| **Guo et al. (2010), OPEN arm (Group A)** | **Open** | 5/16 (31.3%) dancing (highest dancer representation of any subgroup in this study) | 1/16 (6.3%) FHL released for stenosing tenosynovitis | Not reported as discrete figure for this arm | Not reported | Same as endoscopic arm (4/16 had grade I–II anterior impingement) | Same temporal confound as endoscopic arm: open surgery dominant in earlier (pre-2002) part of series | Same rehab protocol as endoscopic arm: no immobilisation, early WB and ROM |
| **Ahn et al. (2013), ENDOSCOPIC arm** | **Endoscopic** | 1/12 (8.3%) dancer | FHL inspected for tenosynovitis in all cases; PRE-EXISTING FHL tendinitis was an EXCLUSION CRITERION for the whole study | Mean 14.6 mo (range 6–24) [endoscopic arm] | Mean 12.6 × 10.4 mm; explicit MRI-measured size, significantly LARGER than arthroscopic arm (p<0.05) | EXCLUDED: combined lateral ankle instability, osteochondral lesion of talus, FHL tendinitis, posttraumatic arthritis, local synovitis/inflammatory arthritis: most rigorously OT-isolated cohort in this review | Single surgeon (J.H.A.), who had completed training courses in both techniques and performed >20 subtalar arthroscopic and posterior endoscopic procedures before the study; no learning-curve analysis | Short-leg splint immobilisation in slightly plantarflexed position; PT/ROM at 48h with ankle boots; stitches removed 2 wk; full WB after 2 wk; RTS training from 6 wk |
| **Ahn et al. (2013), ARTHROSCOPIC arm** | **Subtalar arthroscopy (not open)** | 2/16 (12.5%) dancers | FHL inspected for tenosynovitis in all cases; FHL tendinitis EXCLUSION CRITERION as above | Mean 13.3 mo (range 6–24) [arthroscopic arm] | Mean 11.1 × 8.8 mm; significantly SMALLER than endoscopic arm; 2/16 (12.5%) required intra-op CONVERSION to endoscopy due to large OT/technical difficulty | Same exclusion criteria as endoscopic arm | Same surgeon as endoscopic arm; technique is subtalar arthroscopy, not open excision, and the arm is analysed separately from open arms | Same post-op protocol as endoscopic arm: short-leg splint, PT at 48h, full WB at 2 wk |
| ***OPEN SURGERY – Single arm and dancer-specific cohorts*** | | | | | | | | |
| **Walsh et al. (2020)** | **Open** | 44/44 (100%): dancers only (ballet, jazz, modern, contemporary) | Posteromedial approach used specifically to enable FHL tenolysis/debridement in all cases; pre-existing pathology incidence not separately quantified | Not reported as a discrete cohort figure | Not reported | Not an exclusion-based design; this is a dancer-specific cohort by inclusion, not OT-isolated by exclusion of other pathology | Not reported; single surgeon (J.C.C.); no learning curve analysis; posterolateral approach in the first 16 patients and posteromedial in the subsequent 38 cases (the source mixes patients and cases): technique evolution noted but not analysed as learning curve | CAM boot, partial WB wk 1, WB as tolerated + PT from wk 2; structured 3-phase rehab to 12 wk (detailed protocol in Table 2 of the source study) |
| **Heyer and Rose (2017)** | **Open** | 38/38 (100%) dancers (36/40 ankles ballet specifically, 90%) | 17/40 (42.5%) had FHL symptomatology preoperatively (triggering, clicking, decreased excursion); all 17/17 (100%) relieved postoperatively | Mean 17.6 mo (95% CI 11.7–23.5) | Not reported (no size data; no conversions: open posteromedial approach used throughout, not technique-limited by size) | EXCLUDED: posterolateral or arthroscopic/endoscopic approach, staged procedures, posterior ankle impingement not caused by OT, non-dancers: OT-isolated dancer cohort by design | Not reported; single surgeon (D.J.R.); no learning curve analysis | No cast/splint; removable boot ~2 wk with partial-to-full WB as tolerated; ROM encouraged from post-op day 2–3; formal PT at 2 wk; full dance ~6 wk |

## Expected Direction of Effect

Open vs endoscopic: open → slower return, more complications.

Dancer/ballet cohort: dancer cohorts → longer return, stricter success threshold.

FHL pathology: may predict slower recovery, but treatment may improve final outcome.

Symptom duration: longer duration may worsen outcomes.

Large os trigonum: large OT → technical difficulty, especially subtalar arthroscopy.

Concomitant pathology: heterogeneous pathology → more variable outcomes.

Surgeon experience: early series → longer surgery / more complications.

Rehab protocol: early mobilisation may shorten return.

## Key Observations and Reporting Gaps

Symptom duration is reported as a discrete figure (mean/median months from symptom onset to surgery) in only 8/17 study arms (Heyer and Rose, Ballal both arms, López Valerio, Ahn both arms, Jerosch and Fadel, Morelli). The remaining studies report only the minimum required conservative treatment trial (e.g. '3 months'), which is a related but distinct variable; it reflects protocol threshold rather than actual disease chronicity, and the two should not be conflated when testing this moderator.

Os trigonum size is quantified (MRI-measured, mm) in only one study (Ahn et al.), which is also the only study reporting an intraoperative conversion driven by lesion size/technical difficulty (2/16 arthroscopic cases converted to endoscopy, attributed to larger OT in that subgroup). Other studies use only qualitative descriptors ('large', 'unusually large') for isolated cases without systematic measurement or a denominator and these are noted in the table but cannot support quantitative moderator analysis.

Concomitant pathology handling varies by design rather than by chance: four studies (Nikolopoulos, Ahn, Georgiannos and Bisbinas, Morelli) explicitly excluded concomitant cartilage, instability or soft-tissue pathology, and two were restricted by design (Heyer and Rose, an os trigonum-only dancer cohort; López Valerio, elite soccer players), while others (Sugimoto, Smyth, Guo) included a heterogeneous mix of bony and soft-tissue posterior impingement in the same cohort without subgroup-specific outcome reporting. This is a key source of clinical heterogeneity and should be considered a primary candidate explanation for between-study variability in outcomes, particularly time to RTS and functional scores.

Surgeon experience / learning curve is formally analysed in only one study (Sugimoto et al.), which found a 26-case threshold for proficiency but, importantly, no significant difference in clinical outcomes (AOFAS score, RTS time) between the first and last 10 cases in the series, suggesting that while operative time is learning-curve dependent, clinical outcomes may not be. Guo et al. has an important temporal confound (technique changed from open to endoscopic over the study period, 1994–2007) that conflates surgeon experience/era with surgical technique and should be flagged as a limitation if this study contributes to any technique comparison. Ballal et al. has a structural confound in the opposite direction: two different surgeons each performed only one technique throughout, so technique and surgeon are fully collinear and cannot be statistically separated.

Rehabilitation protocol is well reported across nearly all studies and shows a clear pattern: the endoscopic series and both RCT arms use early weight-bearing/early ROM protocols (no immobilisation, or immobilisation ≤24–48h; Ahn et al. used a short-leg splint with ROM from 48 h and full weight-bearing from 2 weeks, and Morelli et al. kept patients non-weight-bearing for 2 weeks with active ROM from day 2), whereas the open, dancer-specific series (Walsh; Heyer and Rose) use slightly more structured early-phase protection (CAM boot/removable boot for 1–2 weeks) before progressing to early ROM. No study in this review uses prolonged rigid immobilisation (>2 weeks), which limits the ability to test the full expected direction of this moderator (i.e. there is no true 'late mobilisation' comparator group within this dataset).

*Abbreviations: FHL = flexor hallucis longus; mo = months; MRI = magnetic resonance imaging; NR = not reported; OCL = osteochondral lesion; OT = os trigonum; PT = physiotherapy; RCT = randomised controlled trial; ROM = range of motion; RTS = return to sport/dance; WB = weight-bearing.*

**Included studies**

1. Nikolopoulos D, Safos G, Moustakas K, et al. Endoscopic treatment of posterior ankle impingement secondary to os trigonum in recreational athletes. Foot Ankle Orthop 2020;5:2473011420945330.

2. Shimozono Y, Mori D, Mizuno Y, et al. Clinical outcomes of posterior hindfoot endoscopic surgery using 1.9-mm-diameter needle arthroscopy for posterior ankle impingement in athletes. Eur J Orthop Surg Traumatol 2025;35:338.

3. Sugimoto K, Isomoto S, Samoto N, Matsui T, Tanaka Y. Arthroscopic treatment of posterior ankle impingement syndrome: mid-term clinical results and a learning curve. Arthrosc Sports Med Rehabil 2021;3:e1077–86.

4. Smyth NA, Murawski CD, Levine DS, Kennedy JG. Hindfoot arthroscopic surgery for posterior ankle impingement: a systematic surgical approach and case series. Am J Sports Med 2013;41:1869–76.

5. López Valerio V, Seijas R, Alvarez P, et al. Endoscopic repair of posterior ankle impingement syndrome due to os trigonum in soccer players. Foot Ankle Int 2015;36:70–4.

6. Jerosch J, Fadel M. Endoscopic resection of a symptomatic os trigonum. Knee Surg Sports Traumatol Arthrosc 2006;14:1188–93.

7. Morelli F, Mazza D, Serlorenzi P, et al. Endoscopic excision of symptomatic os trigonum in professional dancers. J Foot Ankle Surg 2017;56:22–5.

8. Georgiannos D, Bisbinas I. Endoscopic versus open excision of os trigonum for the treatment of posterior ankle impingement syndrome in an athletic population: a randomised controlled study with 5-year follow-up. Am J Sports Med 2017;45:1388–94.

9. Ballal MS, Roche A, Brodrick A, Williams RL, Calder JDF. Posterior endoscopic excision of os trigonum in professional national ballet dancers. J Foot Ankle Surg 2016;55:927–30.

10. Guo QW, Hu YL, Jiao C, Ao YF, Tian DX. Open versus endoscopic excision of a symptomatic os trigonum: a comparative study of 41 cases. Arthroscopy 2010;26:384–90.

11. Ahn JH, Kim YC, Kim HY. Arthroscopic versus posterior endoscopic excision of a symptomatic os trigonum: a retrospective cohort study. Am J Sports Med 2013;41:1082–9.

12. Walsh KP, Durante EC, Moser BR, Coetzee JC, Stone McGaver R. Surgical outcomes of os trigonum syndrome in dancers: a case series. Orthop J Sports Med 2020;8:2325967120938767.

13. Heyer JH, Rose DJ. Os trigonum excision in dancers via an open posteromedial approach. Foot Ankle Int 2017;38:27–35.
