# Supplementary material for: Defining and detecting poor outcome in heterogeneous surgical cohorts: a proof-of-concept composite distribution- and MCID-based method for study-arm-level evidence synthesis
Source: MethodsX. 2026 Oct 23;17:104232. doi: 10.1016/j.mex.2026.104232 (PMC13742498; doi:10.1016/j.mex.2026.104232)
Supplement: Supplementary file 1 [file mmc1.zip › Supplementary_Table_S2.docx]

# Supplementary Table S2. Risk of bias assessment. Full references for the 13 included studies are listed at the end of Supplementary Table S1.

*Systematic Review: Os Trigonum Surgery in Athletes | Tool: ROBINS-I [1] (non-randomised studies); RoB 2 [2] (RCT). Judgements: ROBINS-I, Low, Moderate, Serious or Critical; RoB 2, Low, Some concerns or High.*

## Part A: ROBINS-I, Non-Randomised Studies

Domains: D1 = Confounding; D2 = Selection of participants; D3 = Classification of intervention; D4 = Deviations from intended intervention; D5 = Missing data; D6 = Measurement of outcomes; D7 = Selection of reported results.

| **Study** | **D1: Confounding** | **D2: Selection of participants** | **D3: Classification of intervention** | **D4: Deviations from intervention** | **D5: Missing data** | **D6: Measurement of outcomes** | **D7: Selection of reported results** | **Overall** | **Key judgements** |
| --- | --- | --- | --- | --- | --- | --- | --- | --- | --- |
| ***Single-arm endoscopic/arthroscopic case series*** | | | | | | | | | |
| **Nikolopoulos et al. (2020)** | **Serious** | **Moderate** | **Low** | **Low** | **Moderate** | **Moderate** | **Moderate** | **Serious** | D1: Retrospective; no adjustment for confounders (sport type, baseline severity, prior steroid injections). D2: Homogeneous inclusion (bony PAIS only) but single-centre. D5: No standardised questionnaire for QoL; informal activity level assessment. D6: Outcome assessors included operating surgeon: measurement bias. D7: Some outcomes (e.g. 2-yr FADI) not reported for all patients. |
| **Shimozono et al. (2025)** | **Moderate** | **Moderate** | **Low** | **Low** | **Low** | **Low** | **Moderate** | **Moderate** | D1: Small, single-centre retrospective cohort; case-mix (ballet vs other) limits generalisability. D2: All patients of senior author: possible referral bias. D7: No preoperative VAS (unconventional rationale); limits pain comparison. |
| **Sugimoto et al. (2021)** | **Moderate** | **Moderate** | **Low** | **Low** | **Low** | **Low** | **Moderate** | **Moderate** | D1: Heterogeneous PAIS aetiology (OT, talar process, soft tissue): confounds subgroup analysis. D6: Follow-up by operating surgeon pair. D7: Pre-2013 cohort did not complete SAFE-Q; outcomes incomplete for ~half the series. |
| **Smyth et al. (2013)** | **Serious** | **Moderate** | **Low** | **Low** | **Low** | **Low** | **Moderate** | **Serious** | D1: Heterogeneous population (soft tissue + bony PAIS); no attempt to adjust. D2: All from 2 senior surgeons at specialist centre. D6: Assessors not independent. D7: 1 patient FU only 14 months: marginal inclusion. Outcomes in the review are extracted for the 16 athletes with an os trigonum. |
| **López Valerio et al. (2015)** | **Moderate** | **Moderate** | **Low** | **Low** | **Low** | **Low** | **Moderate** | **Moderate** | D1: Homogeneous (soccer, Tegner 9): internally consistent but limits generalisability. D5: VAS measured only at RTS (not standardised post-op time). D7: Return to sport defined as Tegner level: well-specified. |
| **Jerosch and Fadel (2006)** | **Serious** | **Serious** | **Low** | **Low** | **Low** | **Moderate** | **Serious** | **Serious** | D1: Very small N; no confounding adjustment. D2: Referral centre, 3/10 professionals: selection bias. D6: AOFAS only; no VAS pre-op or standardised ROM. D7: Short minimum FU (6 months); limited reporting of residual symptoms. |
| **Morelli et al. (2017)** | **Moderate** | **Moderate** | **Low** | **Low** | **Low** | **Moderate** | **Moderate** | **Moderate** | D1: Small, single-centre retrospective series; homogeneous professional ballet dancers (limits case-mix confounding) but no adjustment for confounders. D2: Professional dancers treated 2010–2015; 2 of 14 excluded for a combined osteochondral lesion of the talus. D5: All 12 included patients assessed at final follow-up. D6: Outcomes assessed by the treating team, not blinded; return defined as the start of dancing, level not specified. D7: No protocol available; all outcomes listed in the Methods are reported. |
| ***Single-arm open surgery case series*** | | | | | | | | | |
| **Walsh et al. (2020)** | **Moderate** | **Moderate** | **Low** | **Low** | **Moderate** | **Moderate** | **Moderate** | **Moderate** | D1: All dancers; single surgeon: reduced confounding but may not generalise. D5: Outcome at 'last FU' (highly variable 12–102 mo) rather than discrete timepoint: introduces variability. D6: PROs self-reported; satisfaction 83% (subjective). D7: No formal functional score (FFI-R used but not validated for PAIS). |
| **Heyer and Rose (2017)** | **Moderate** | **Moderate** | **Low** | **Low** | **Moderate** | **Moderate** | **Moderate** | **Moderate** | D1: Single surgeon, single technique: reduces technical variability. D5: 15/55 lost to FU (retrospective telephone interview). D6: Retrospective pain recall subject to recall bias. D7: 'Time to return to dance' and 'pain-free dance' both reported: clear outcome hierarchy. |
| ***Non-randomised comparative studies (ROBINS-I)*** | | | | | | | | | |
| **Ballal et al. (2016)** | **Serious** | **Serious** | **Low** | **Moderate** | **Low** | **Moderate** | **Serious** | **Serious** | D1: Two different surgeons, different patient ages (open mean 17.6 vs endoscopic 20.4 yr, p=0.015): significant confounding. D2: Technique allocated by surgeon preference, not random: selection bias critical. D6: No formal PROMs; time to RTS is primary comparator. D7: FHL decompression rate differs markedly (59% open vs 15% endoscopic, p=0.016): this is now a formally tracked outcome in this review, but surgeon and technique are fully collinear here, so the FHL finding cannot be attributed to technique versus surgeon threshold for treating FHL endoscopically. |
| **Guo et al. (2010)** | **Serious** | **Serious** | **Low** | **Moderate** | **Moderate** | **Moderate** | **Moderate** | **Serious** | D1: Different professional distribution (dancers in open, soccer in endoscopic): major confounder for RTS time. D2: Technique evolved over time (open pre-2002, then mixed, endoscopic post-2006): temporal confounding, also affects FHL decompression rate comparison (1/16 open vs 2/25 endoscopic) since surgeon experience and technique era are conflated. D7: Pre-operative VAS not collected: hampers pain comparison. |
| **Ahn et al. (2013)** | **Serious** | **Serious** | **Low** | **Moderate** | **Low** | **Low** | **Moderate** | **Serious** | D1: Os trigonum larger in endoscopic group (p<0.05): selection confounds; the authors state that technique was selected without preference, but the size difference suggests allocation may not have been independent of lesion size. D2: Not randomised; technique selection without blinding. Critical confounder for technical feasibility outcomes. Note: pre-existing FHL tendinitis was an exclusion criterion for this study, so FHL pathology findings are not comparable with other included studies. Compares posterior endoscopy with subtalar arthroscopy (both arthroscopic). |

## Part B: RoB 2, Randomised Controlled Trial

Domains: B1 = Randomisation process; B2 = Deviations from intended intervention; B3 = Missing outcome data; B4 = Measurement of outcome; B5 = Selection of reported result.

| **Study** | **B1: Randomisation process** | **B2: Deviations from intended intervention** | **B3: Missing outcome data** | **B4: Measurement of outcome** | **B5: Selection of reported result** | **Overall** | **Key judgements** |
| --- | --- | --- | --- | --- | --- | --- | --- |
| **Georgiannos and Bisbinas (2017)** | **Low** | **Some concerns** | **Low** | **Some concerns** | **Low** | **Some concerns** | B1: Computer-generated randomisation: LOW. B2: Nature of surgical intervention means blinding of participants and surgeons is impossible; however, all arms received same post-operative protocol: SOME CONCERNS. B3: No loss to FU stated; 5-year follow-up complete: LOW. B4: Outcome assessors not blinded to intervention; AOFAS scored by treating team: SOME CONCERNS. B5: Both primary and secondary outcomes reported per protocol; no evidence of selective reporting: LOW. Overall: SOME CONCERNS (driven by inherent inability to blind in surgical trials). |

*Notes: The ROBINS-I overall rating is at least as severe as the most severe domain rating. For surgical studies, blinding of participants and surgeons is structurally impossible; this is acknowledged but not penalised under ROBINS-I D4 provided deviations from intended intervention were not differential. Abbreviations: FHL = flexor hallucis longus; FU = follow-up; NR = not reported; OT = os trigonum; PAIS = posterior ankle impingement syndrome; PRO = patient-reported outcome; RCT = randomised controlled trial; RoB 2 = Risk of Bias tool 2 (Cochrane); ROBINS-I = Risk Of Bias In Non-randomised Studies of Interventions; RTS = return to sport; VAS = visual analogue scale.*

**References**

[1] J.A.C. Sterne, M.A. Hernán, B.C. Reeves, et al., ROBINS-I: a tool for assessing risk of bias in non-randomised studies of interventions, BMJ 355 (2016) i4919.

[2] J.A.C. Sterne, J. Savović, M.J. Page, et al., RoB 2: a revised tool for assessing risk of bias in randomised trials, BMJ 366 (2019) l4898.
