# Supplementary material for: Defining and detecting poor outcome in heterogeneous surgical cohorts: a proof-of-concept composite distribution- and MCID-based method for study-arm-level evidence synthesis
Source: MethodsX. 2026 Oct 23;17:104232. doi: 10.1016/j.mex.2026.104232 (PMC13742498; doi:10.1016/j.mex.2026.104232)
Supplement: Supplementary file 1 [file mmc1.zip › Supplementary_Table_S3.docx]

# Supplementary Table S3. GRADE evidence profile. Full references for the 13 included studies are listed at the end of Supplementary Table S1.

*Systematic Review: Os Trigonum Surgery in Athletes | Certainty levels: High, Moderate, Low, Very low; each serious concern lowers certainty by one level (−1) and each very serious concern by two (−2). Starting certainty: High for the RCT; Low for non-randomised studies (observational starting point), in line with GRADE guidance [1]. Downgrading criteria: Risk of bias (RoB), Inconsistency, Indirectness, Imprecision, Publication bias. Upgrading criteria: Large effect, Dose-response, All plausible confounders favour the effect.*

| **Outcome** | **Studies (N)** | **Study design** | **Risk of bias** | **Inconsistency** | **Indirectness** | **Imprecision** | **Publication bias** | **Upgrade factors** | **GRADE certainty** | **Effect (summary)** |
| --- | --- | --- | --- | --- | --- | --- | --- | --- | --- | --- |
| ***ENDOSCOPIC / ARTHROSCOPIC OS TRIGONUM EXCISION IN ATHLETES: All study designs pooled*** | | | | | | | | | | |
| **1. Failed return to prior sport/dance level** | 11 endoscopic arms (N = 277) | Non-randomised (case series, cohort, 1 RCT endoscopic arm) | Serious risk of bias: retrospective design, no blinding; operating surgeon assessing return; selection bias in comparator studies. −1 | Not serious: all studies report 0–6.2% failure; directionally consistent. 0 | Not serious: outcomes specifically in athletic populations. 0 | Serious: small samples, single-arm studies; 95% CIs wide in small arms (upper limits up to 27.8%). −1 | Not detected: case series unlikely to under-report failures but small N limits detection. 0 | None | **Very low** | Pooled failure rate: ~2% (range 0–6.2%). Most studies report 0/N failures. Single RCT endoscopic arm: 0/26. 1 study (López Valerio) 1/20 required revision. Return after endoscopic excision was near-universal. |
| **2. Time to full return to sport/dance (weeks)** | 11 endoscopic arms reporting a time | Non-randomised (case series, 1 RCT endoscopic arm) | Serious: retrospective; RTS defined inconsistently (training vs competition vs full prior level); operating team often confirmed return. −1 | Not serious: full or prior-level return 6.0–13.6 wk across arms; variation partly reflects differing definitions of return. 0 | Serious: dancer cohorts not directly comparable to mixed athlete cohorts. −1 | Not serious: sufficient sample sizes for point estimates; ranges adequately reported. 0 | Undetected: publication bias possible (positive results more likely published) but both early and late RTS reported. 0 | None | **Very low** | Full or prior-level return 6.0–13.6 wk (7 arms; earlier training or limited return in 4 of these, 2.8–5.0 wk); return at an unspecified level 8.0–9.1 wk (4 further arms). Definitions of return differ between studies (Supplementary Table S1). RCT: 7.12 wk endoscopic vs 11.54 wk open (p < 0.001). |
| **3. Any complication** | 11 endoscopic arms (N = 306) | Non-randomised | Serious: complication definitions vary; retrospective ascertainment; short FU in some studies. −1 | Not serious: complication rates consistently low (0–9.1%); sural nerve dysaesthesia most common. 0 | Not serious: complications are objective events. 0 | Serious: low event rates; studies underpowered for rare events; 95% CI wide. −1 | Not detected: publication bias unlikely for complications (these are reported even in positive case series). 0 | None | **Very low** | Endoscopic: 0–9.1% (median 5.0% across arms). Needle arthroscopy: 0/13. Predominantly transient neurapraxia. No neurovascular injury described as permanent in endoscopic arms (one tibial-nerve sensory loss of unstated duration in Guo et al.). |
| **4. Neurological complication** | 11 endoscopic arms (N = 306) | Non-randomised | Serious: underdiagnosis possible (informal examination); retrospective. −1 | Not serious: consistent (transient sural neurapraxia is the recognised risk). 0 | Not serious. 0 | Serious: very low event count; cannot estimate rate with precision. −1 | Not detected. 0 | None | **Very low** | Endoscopic: 0–8.3% neurological, mostly transient sural neurapraxia; one tibial-nerve sensory loss of unstated duration (Guo); none described as permanent in any endoscopic arm (0/306). |
| **5. Wound complication** | 11 endoscopic arms (N = 306) | Non-randomised | Serious. −1 | Not serious: wound complications rare across all endoscopic series; 1 study reports portal drainage related to prior steroid injections. 0 | Not serious. 0 | Serious: very low event count. −1 | Not detected. 0 | None | **Very low** | Endoscopic: 0–4.5% wound issues: prolonged portal drainage (1) [Nikolopoulos], 3 superficial skin burns with monopolar cautery in the early series [Sugimoto], 1 infection requiring washout [Smyth]. |
| **6. Residual symptoms / reoperation** | 11 endoscopic arms (N = 306) | Non-randomised | Serious: residual symptoms self-reported; variable definitions (pain, functional limitation, lower activity level). −1 | Not serious: residual symptom rates consistently low (<10%) across studies. 0 | Not serious. 0 | Serious: wide variation in FU duration; some reoperations may be missed with short FU. −1 | Not detected. 0 | None | **Very low** | Endoscopic: 0–6.2% lower activity level; 1.2–5% requiring reoperation. No study reports >1 formal revision. Sugimoto: 1 scar → 2nd procedure. Nikolopoulos: 1 arthrofibrosis treated operatively. Smyth: 1 washout for infection. Georgiannos and López Valerio: 1 revision each for persistent pain. |
| **7. Functional score (AOFAS / FAOS / SAFE-Q / JSSF: final follow-up)** | 10 endoscopic arms | Non-randomised | Serious: assessors frequently not independent of treating surgeon; retrospective data. −1 | Not serious: all studies show large, significant improvement regardless of score used. 0 | Not serious: directly relevant population and intervention. 0 | Not serious: all studies report statistically significant improvement (p<0.001). 0 | Not detected. 0 | None | **Very low** | AOFAS: pre-op 39–80, final 87–98. FAOS (os trigonum athletes): 60.9 → 89.3. JSSF: 79 → 98. SAFE-Q pain: 70.6 → 97.7. All clinically meaningful improvements. All p<0.001. |
| **8. FHL pathology (reported as % cohort with FHL symptoms/tenosynovitis, or % requiring FHL decompression)** | 10 studies (N ≈ 200) | Non-randomised (case series, cohort, RCT both arms) | Very serious: marked variation in how FHL pathology is ascertained and reported across studies: some report baseline symptomatic FHL tenosynovitis (cohort characteristic), others report rate of surgical FHL decompression (procedural outcome), others describe routine FHL inspection without quantifying pathology. These are not the same construct. −2 | Serious: reported rates range from 8% to 100% depending on definition and population (e.g. inclusion criteria requiring FHL tenosynovitis vs incidental finding). −1 | Not serious: directly relevant population. 0 | Serious: most individual study estimates based on small samples. −1 | Not detected. 0 | None | **Very low** | Endoscopic series: 8–100% depending on definition (Shimozono 100% by inclusion criterion; Guo 8% requiring decompression). Ballal: FHL decompression required in 15.4% endoscopic vs 59.1% open (p=0.016), but technique and surgeon are fully confounded in this study. Ahn et al. excluded pre-existing FHL tendinitis entirely, limiting comparability. |
| ***ENDOSCOPIC vs OPEN: Comparative Evidence (RCT + Non-Randomised Comparison Studies)*** | | | | | | | | | | |
| **1. Failed return to prior sport/dance level (endoscopic vs open)** | 3 open-vs-endoscopic studies (N = 128); Ahn et al. reported separately | 1 RCT + 2 non-randomised comparative | Very serious: only 1 RCT; non-randomised comparisons have critical confounding (different surgeons, patient age, sport mix, lesion size). −2 | Serious: consistent direction (endoscopic ≤ open) but magnitudes differ across studies; open arm failure rates 0–4.5%. −1 | Not serious: same indication and population. 0 | Serious: 1 RCT (N = 52); too few failure events for any estimate. −1 | Undetected. 0 | None | **Very low** | RCT: 0% both arms at 5 yr. Ballal: 0% endoscopic vs 4.5% open (1/22, unrelated hip injury). Guo: 0% both. No statistically significant difference. Failure rates are very low for both approaches. (Ahn, endoscopy vs subtalar arthroscopy: 0% both.) |
| **2. Time to full RTS: endoscopic vs open** | 3 open-vs-endoscopic studies (N = 128) | 1 RCT + 2 non-randomised | Serious: RCT at some concerns (unblinded; timing reported by treating team); non-randomised comparisons confounded by surgeon [Ballal] and era [Guo]. −1 | Not serious: all 3 studies favour endoscopic (earlier RTS); direction consistent. 0 | Not serious. 0 | Serious: single RCT of 52 patients, below the optimal information size (about 400) [2] for a continuous outcome; pooled estimate relies on imputed SDs for two studies. −1 | Possible: endoscopic may be systematically reported by its proponents. 0 | Large effect (upgrade considered but not applied without adjustment for confounders) | **Low** | RCT: 7.12 vs 11.54 wk (MD 4.42, 95% CI 2.69 to 6.15; p < 0.001). Ballal: 9.8 vs 14.9 wk (p = 0.001). Guo: 6.0 vs 11.9 wk (p < 0.001). Pooled MD 4.92 wk (3.27 to 6.57; I² = 0%; prediction interval 2.33 to 7.51), imputed SDs. Consistent advantage for endoscopic approach. (Ahn, endoscopy vs subtalar arthroscopy: 8.0 vs 7.5 wk, NS; not pooled.) |
| **3. Any complication: endoscopic vs open** | 3 open-vs-endoscopic studies (N = 128) | 1 RCT + 2 non-randomised | Serious: complication ascertainment retrospective in non-RCT arms. −1 | Not serious: both estimable studies favour endoscopic; one study recorded no events in either arm. 0 | Not serious. 0 | Very serious: 10 events in the two estimable studies; RCT OR 7.50 (95% CI 0.83 to 67.49); pooled OR 5.59 (1.10 to 28.35); optimal information size (about 255 per group) not met (64 per group available). −2 | Not detected. 0 | None | **Very low** | RCT: 1/26 (3.8%) endoscopic vs 6/26 (23.1%) open; OR 7.50 (0.83 to 67.49); Fisher's exact p = 0.099 on published counts (trial reports p < 0.001). Ballal: 0/13 vs 0/22. Guo: 1/25 (4%) vs 2/16 (12.5%). Pooled Mantel–Haenszel OR 5.59 (1.10 to 28.35). Direction favours endoscopic; magnitude uncertain. (Ahn, vs subtalar arthroscopy: 1/12 vs 1/16; not pooled.) |
| **4. Neurological complication: endoscopic vs open** | 3 open-vs-endoscopic studies (N = 128) | 1 RCT + 2 non-randomised | Serious: retrospective ascertainment in non-RCT. −1 | Not serious: endoscopic consistently associated with lower neurological complication rate. 0 | Not serious. 0 | Very serious: 5 events in total across the three studies. −2 | Not detected. 0 | None | **Very low** | RCT: 0% endoscopic vs 11.5% open (2 transient, 1 permanent sural nerve injury). Guo: 4% (medial heel numbness) vs 6.3% (permanent sural nerve loss). Ballal: none. Total 1/64 vs 4/64 (Fisher's exact p = 0.37). No neurological deficit described as permanent after endoscopic excision in any arm (0/306; Guo endoscopic sensory loss of unstated duration) vs 2/148 after open; too few events to exclude chance. |
| **5. FHL decompression rate: endoscopic vs open** | 2 studies (N = 76) | 2 non-randomised comparative (Ballal et al.; Guo et al.) | Very serious: both studies are confounded: surgeon and technique fully collinear in Ballal et al., era and technique in Guo et al.; any difference cannot be attributed to technique. −2 | Serious: the two studies point in opposite directions (Ballal 15.4% endoscopic vs 59.1% open; Guo 8.0% vs 6.3%). −1 | Serious: dancer-only population in Ballal et al.; Guo et al. not restricted to athletes. −1 | Serious: small samples (N = 35 and 41) and few decompressions in Guo et al. (3 in total). −1 | Not detected. 0 | None | **Very low** | Ballal: 2/13 (15.4%) endoscopic vs 13/22 (59.1%) open required FHL decompression (p = 0.016). Guo: 2/25 (8.0%) vs 1/16 (6.3%). The two studies disagree and both are confounded; the Ballal difference may reflect one surgeon's threshold for treating a tendon in full view rather than a true difference in pathology between techniques. |

## GRADE Summary of Findings

For endoscopic excision of os trigonum in athletes (single-arm data from all endoscopic arms), the certainty of evidence is VERY LOW for every outcome: the evidence is observational and was downgraded for risk of bias and, for most outcomes, imprecision or indirectness. Across the eleven endoscopic arms, return to prior sport level was near-universal (93.8–100%), complication rates were low (0–9.1%), and recovery was rapid, although return to sport was defined differently across studies. For the direct comparison of endoscopic with open excision, the single RCT, supported in direction by two non-randomised comparisons, provides LOW certainty that endoscopic surgery shortens return to sport. Certainty that it reduces complications is VERY LOW: the estimate is imprecise (10 events in the two estimable studies; RCT OR 7.50, 95% CI 0.83 to 67.49) and the optimal information size is not met. No nerve injury described as permanent followed endoscopic excision (one endoscopic sensory loss in Guo et al. was of unstated duration), but this rests on very few events (VERY LOW certainty). Evidence on FHL pathology is of VERY LOW certainty: reporting is inconsistent across studies (cohort characteristic vs procedural outcome vs unquantified routine inspection), and the two studies reporting FHL decompression by technique (Ballal et al.; Guo et al.) disagree and are both confounded, by surgeon and by era respectively.

*Abbreviations: AOFAS = American Orthopaedic Foot & Ankle Society hindfoot score; CRPS = complex regional pain syndrome; FADI = Foot & Ankle Disability Index; FAOS = Foot and Ankle Outcome Score; FHL = flexor hallucis longus; GRADE = Grading of Recommendations, Assessment, Development and Evaluation; JSSF = Japanese Society for Surgery of the Foot scale; NS = not significant; PAIS = posterior ankle impingement syndrome; RCT = randomised controlled trial; RoB = risk of bias; RTS = return to sport or dance; SAFE-Q = Self-Administered Foot Evaluation Questionnaire; wk = week(s).*

**References**

[1] G.H. Guyatt, A.D. Oxman, G.E. Vist, et al., GRADE: an emerging consensus on rating quality of evidence and strength of recommendations, BMJ 336 (2008) 924–926. https://doi.org/10.1136/bmj.39489.470347.AD.

[2] G.H. Guyatt, A.D. Oxman, R. Kunz, et al., GRADE guidelines 6. Rating the quality of evidence: imprecision, J. Clin. Epidemiol. 64 (2011) 1283–1293. https://doi.org/10.1016/j.jclinepi.2011.01.012.
