# Supplementary material for: Defining and detecting poor outcome in heterogeneous surgical cohorts: a proof-of-concept composite distribution- and MCID-based method for study-arm-level evidence synthesis
Source: MethodsX. 2026 Oct 23;17:104232. doi: 10.1016/j.mex.2026.104232 (PMC13742498; doi:10.1016/j.mex.2026.104232)
Supplement: Supplementary file 1 [file mmc1.zip › Supplementary_Table_S4.docx]

**Supplementary Table S4. Quantitative synthesis: per-study and pooled estimates, sensitivity analyses and arm-level event rates. Full references for the 13 included studies are listed at the end of Supplementary Table S1.**

**Part A. Time to full or prior-level return to sport or dance: mean difference (open minus endoscopic, weeks)**

| **Study / analysis** | **Open mean (SD), n** | **Endoscopic mean (SD), n** | **MD (95% CI)** | **Weight** | **SD source** |
| --- | --- | --- | --- | --- | --- |
| Georgiannos and Bisbinas (RCT) | 11.54 (3.89), 26 | 7.12 (2.25), 26 | 4.42 (2.69 to 6.15) | 46.7% | Reported |
| Ballal et al. | 14.9 (2.88), 22 | 9.8 (2.87), 13 | 5.10 (3.13 to 7.07) | 35.9% | Imputed from range (Wan et al. [1]) |
| Guo et al. | 11.9 (5.65), 16 | 6.0 (1.53), 25 | 5.90 (3.07 to 8.73) | 17.4% | Imputed from range (Wan et al. [1]) |
| Random-effects (REML, Hartung–Knapp), 3 studies |  |  | 4.92 (3.27 to 6.57) | 100% | τ² = 0; I² = 0%; Q = 0.81, df = 2, p = 0.67; 95% prediction interval 2.33 to 7.51 (t, k − 1 df) |
| Primary estimate: RCT alone (reported SDs) |  |  | 4.42 (2.69 to 6.15) |  |  |
| Sensitivity: excluding Guo et al. (not athlete-restricted) |  |  | 4.72 (0.43 to 9.00) |  | 2 studies; Hartung–Knapp |
| Sensitivity: fixed effect (inverse variance) |  |  | 4.92 (3.74 to 6.10) |  |  |
| Not pooled: Ahn et al., subtalar arthroscopy minus endoscopy | 7.5 (1.6), 16 | 8.0 (1.8), 12 | −0.50 (−1.79 to 0.79) |  | Early milestone (start of running and jumping) |

**Part B. Any complication: odds ratio (OR > 1 = more complications after open excision)**

| **Study / analysis** | **Open events/N** | **Endoscopic events/N** | **OR (95% CI)** | **Fisher's exact p (Yates χ² p)** | **Comment** |
| --- | --- | --- | --- | --- | --- |
| Georgiannos and Bisbinas (RCT) | 6/26 | 1/26 | 7.50 (0.83 to 67.49) | 0.099 (0.104) | Trial report gives p < 0.001, not reproducible from counts |
| Guo et al. | 2/16 | 1/25 | 3.43 (0.28 to 41.32) | 0.550 (0.686) | Era confound |
| Ballal et al. | 0/22 | 0/13 | Not estimable | – | No events in either arm |
| Mantel–Haenszel (fixed), 2 studies | 8/42* | 2/51* | 5.59 (1.10 to 28.35) | p = 0.038 | I² = 0%; Q = 0.21, df = 1; 10 events |
| Sensitivity: Peto OR, 2 studies |  |  | 4.47 (1.20 to 16.66) |  |  |
| Sensitivity: including Ahn et al. (subtalar arthroscopy as comparator) |  |  | 3.53 (0.92 to 13.57) |  |  |
| Not pooled: Ahn et al., subtalar arthroscopy vs endoscopy | 1/16 | 1/12 | 0.73 (0.04 to 13.05) | 1.000 (1.000) | Both arthroscopic |
| Information size |  |  |  |  | About 255 patients per group needed to detect 12.2% vs 5.2% (80% power, two-sided α 0.05); 64 per group available |

**Counts in the two estimable studies. Across the three open-versus-endoscopic studies, 8/64 open and 2/64 endoscopic.*

**Part C. Neurological complications**

| **Comparison** | **Open** | **Endoscopic** | **Fisher's exact p** |
| --- | --- | --- | --- |
| Three open-versus-endoscopic studies | 4/64 | 1/64 | 0.37 |
| All arms (unweighted, descriptive) | 6/148 (4.1%) | 6/306 (2.0%) | 0.22 |
| Permanent nerve injury, all arms | 2/148 | 0/306 | 0.11 |

**Part D. Arm-level event rates with 95% confidence intervals (Wilson)**

| **Study arm** | **Technique** | **Unit** | **Failed return n/N** | **% (95% CI)** | **Any complication n/N** | **% (95% CI)** |
| --- | --- | --- | --- | --- | --- | --- |
| Nikolopoulos et al. | Endoscopic | Patients | 5/81 | 6.2 (2.7 to 13.6) | 5/81 | 6.2 (2.7 to 13.6) |
| Shimozono et al. | Endoscopic | Patients | 0/13 | 0.0 (0.0 to 22.8) | 0/13 | 0.0 (0.0 to 22.8) |
| Sugimoto et al. | Endoscopic | OT ankles (failure); whole-cohort ankles (complications) | 0/49 | 0.0 (0.0 to 7.3) | 4/72 | 5.6 (2.2 to 13.4) |
| Smyth et al. | Endoscopic | OT athletes (failure); whole cohort (complications) | 0/16 | 0.0 (0.0 to 19.4) | 2/22 | 9.1 (2.5 to 27.8) |
| López Valerio et al. | Endoscopic | Patients | 1/20 | 5.0 (0.9 to 23.6) | 1/20 | 5.0 (0.9 to 23.6) |
| Jerosch and Fadel | Endoscopic | Patients | 0/10 | 0.0 (0.0 to 27.8) | 0/10 | 0.0 (0.0 to 27.8) |
| Morelli et al. | Endoscopic | Patients | 0/12 | 0.0 (0.0 to 24.2) | 1/12 | 8.3 (1.5 to 35.4) |
| Georgiannos and Bisbinas (endoscopic) | Endoscopic | Patients | 0/26 | 0.0 (0.0 to 12.9) | 1/26 | 3.8 (0.7 to 18.9) |
| Georgiannos and Bisbinas (open) | Open | Patients | 0/26 | 0.0 (0.0 to 12.9) | 6/26 | 23.1 (11.0 to 42.1) |
| Ballal et al. (endoscopic) | Endoscopic | Patients | 0/13 | 0.0 (0.0 to 22.8) | 0/13 | 0.0 (0.0 to 22.8) |
| Ballal et al. (open) | Open | Patients | 1/22 | 4.5 (0.8 to 21.8) | 0/22 | 0.0 (0.0 to 14.9) |
| Guo et al. (endoscopic) | Endoscopic | Patients | 0/25 | 0.0 (0.0 to 13.3) | 1/25 | 4.0 (0.7 to 19.5) |
| Guo et al. (open) | Open | Patients | 0/16 | 0.0 (0.0 to 19.4) | 2/16 | 12.5 (3.5 to 36.0) |
| Ahn et al. (endoscopic) | Endoscopic | Patients | 0/12 | 0.0 (0.0 to 24.3) | 1/12 | 8.3 (1.5 to 35.4) |
| Ahn et al. (subtalar arthroscopy) | Subtalar arthroscopy | Patients | 0/16 | 0.0 (0.0 to 19.4) | 1/16 | 6.3 (1.1 to 28.3) |
| Walsh et al. | Open | Patients | 0/44 | 0.0 (0.0 to 8.0) | 6/44 | 13.6 (6.4 to 26.7) |
| Heyer and Rose | Open | Ankles (40) in 38 patients | 2/37 | 5.4 (1.5 to 17.7) | 4/40 | 10.0 (4.0 to 23.1) |
| Endoscopic, all arms |  |  | 6/277 | 2.2 (1.0 to 4.6) | 16/306 | 5.2 (3.2 to 8.3) |
| Open, all arms |  |  | 3/145 | 2.1 (0.7 to 5.9) | 18/148 | 12.2 (7.8 to 18.4) |
| Subtalar arthroscopy |  |  | 0/16 | 0.0 (0.0 to 19.4) | 1/16 | 6.3 (1.1 to 28.3) |

*Crude totals combine different case mixes, surgeons and complication definitions and are descriptive only.*

**Part E. Sensitivity of the exploratory composite classification to the component B threshold**

| **Study arm** | **Technique** | **A** | **D** | **B: common threshold 14.55 wk (primary)** | **Score** | **B: technique-specific (11.80 endoscopic; 16.89 open)** | **Score** | **B: best available milestone (13.30 wk)** | **Score** |
| --- | --- | --- | --- | --- | --- | --- | --- | --- | --- |
| Nikolopoulos et al. | Endoscopic | 1 | 1 | – | 2 | – | 2 | 0 | 2 |
| Shimozono et al. | Endoscopic | 0 | 0 | 0 | 0 | 0 | 0 | 0 | 0 |
| Sugimoto et al. | Endoscopic | 0 | 1 | 0 | 1 | 1 | 2 | 1 | 2 |
| Smyth et al. | Endoscopic | 0 | 1 | 0 | 1 | 1 | 2 | 0 | 1 |
| López Valerio et al. | Endoscopic | 1 | 1 | 0 | 2 | 0 | 2 | 0 | 2 |
| Jerosch and Fadel | Endoscopic | 0 | 0 | – | 0 | – | 0 | 0 | 0 |
| Morelli et al. | Endoscopic | 0 | 0 | – | 0 | – | 0 | 0 | 0 |
| Georgiannos and Bisbinas (endoscopic) | Endoscopic | 0 | 1 | 0 | 1 | 0 | 1 | 0 | 1 |
| Georgiannos and Bisbinas (open) | Open | 0 | 1 | 0 | 1 | 0 | 1 | 0 | 1 |
| Ballal et al. (endoscopic) | Endoscopic | 0 | 0 | 0 | 0 | 0 | 0 | 0 | 0 |
| Ballal et al. (open) | Open | 1 | 0 | 1 | 2 | 0 | 1 | 1 | 2 |
| Guo et al. (endoscopic) | Endoscopic | 0 | 1 | 0 | 1 | 0 | 1 | 0 | 1 |
| Guo et al. (open) | Open | 0 | 1 | 0 | 1 | 0 | 1 | 0 | 1 |
| Ahn et al. (endoscopic) | Endoscopic | 0 | 1 | – | 1 | – | 1 | 0 | 1 |
| Ahn et al. (subtalar arthroscopy) | Subtalar | 0 | 1 | – | 1 | – | 1 | 0 | 1 |
| Walsh et al. | Open | 0 | 1 | – | 1 | – | 1 | – | 1 |
| Heyer and Rose | Open | 1 | 1 | 1 | 3 | 1 | 3 | 1 | 3 |

*Component C was not met in any arm. Common threshold: mean 10.68 + SD 3.87 weeks across the 11 arms reporting a full or prior-level return. Technique-specific: endoscopic mean 8.77 + SD 3.03 (7 arms); open mean 14.01 + SD 2.88 (4 arms). Best available milestone: mean 9.92 + SD 3.38 across 16 arms using the best available figure. –, not assessable. The same analyses, with all other specifications, are in Supplementary Table S5. Arms with a poor-outcome profile (score ≥ 2): common threshold, 2 endoscopic and 2 open; technique-specific, 4 endoscopic and 1 open; best available milestone, 3 endoscopic and 2 open.*

**References**

[1] X. Wan, W. Wang, J. Liu, T. Tong, Estimating the sample mean and standard deviation from the sample size, median, range and/or interquartile range, BMC Med. Res. Methodol. 14 (2014) 135.
