# Supplementary material for: Defining and detecting poor outcome in heterogeneous surgical cohorts: a proof-of-concept composite distribution- and MCID-based method for study-arm-level evidence synthesis
Source: MethodsX. 2026 Oct 23;17:104232. doi: 10.1016/j.mex.2026.104232 (PMC13742498; doi:10.1016/j.mex.2026.104232)
Supplement: Supplementary file 1 [file mmc1.zip › Supplementary_Table_S5.docx]

**Supplementary Table S5. Sensitivity analyses of the study-arm-level poor-outcome classification in the worked example (17 study arms from 13 studies).**

*All values are reproduced by Supplementary File S1 (base R). Full references for the 13 included studies are listed at the end of Supplementary Table S1; other references are listed at the end of this table. Arm sizes are participants, except Sugimoto et al. (50 ankles in the os trigonum subgroup) and Heyer and Rose (38 dancers, 40 ankles).*

**Part A. Classification under each specification**

| **Family** | **Specification** | **Arms classified (n)** | **E / O / S** | **Changed vs primary** | **κ** | **Arms classified** |
| --- | --- | --- | --- | --- | --- | --- |
| Primary | Primary: ≥ 2 of 4; B = mean + 1 SD (common) | 4 | 2 / 2 / 0 | – | – | Nikolopoulos et al.; López Valerio et al.; Ballal et al. (open); Heyer and Rose |
| Classification rule | ≥ 1 of 4 | 13 | 7 / 5 / 1 | 9 | 0.17 | Nikolopoulos et al.; Sugimoto et al.; Smyth et al.; López Valerio et al.; Georgiannos and Bisbinas (endoscopic); Georgiannos and Bisbinas (open); Ballal et al. (open); Guo et al. (endoscopic); Guo et al. (open); Ahn et al. (endoscopic); Ahn et al. (subtalar arthroscopy); Walsh et al.; Heyer and Rose |
| Classification rule | ≥ 3 of 4 | 1 | 0 / 1 / 0 | 3 | 0.34 | Heyer and Rose |
| Component B threshold | B = mean + 0.5 SD (12.61 wk) | 5 | 3 / 2 / 0 | 1 | 0.85 | Nikolopoulos et al.; Sugimoto et al.; López Valerio et al.; Ballal et al. (open); Heyer and Rose |
| Component B threshold | B = mean + 1.5 SD (16.48 wk) | 3 | 2 / 1 / 0 | 1 | 0.82 | Nikolopoulos et al.; López Valerio et al.; Heyer and Rose |
| Component B threshold | B = mean + 2.0 SD (18.42 wk) | 3 | 2 / 1 / 0 | 1 | 0.82 | Nikolopoulos et al.; López Valerio et al.; Heyer and Rose |
| Component B threshold | B = sample-size-weighted mean + 1 SD (15.61 wk) | 3 | 2 / 1 / 0 | 1 | 0.82 | Nikolopoulos et al.; López Valerio et al.; Heyer and Rose |
| Component B threshold | B = 75th percentile of arm means (12.75 wk) | 5 | 3 / 2 / 0 | 1 | 0.85 | Nikolopoulos et al.; Sugimoto et al.; López Valerio et al.; Ballal et al. (open); Heyer and Rose |
| Component B threshold | B = 90th percentile of arm means (14.90 wk) | 3 | 2 / 1 / 0 | 1 | 0.82 | Nikolopoulos et al.; López Valerio et al.; Heyer and Rose |
| Component B threshold | B = fixed 12 wk (not data-derived) | 5 | 3 / 2 / 0 | 1 | 0.85 | Nikolopoulos et al.; Sugimoto et al.; López Valerio et al.; Ballal et al. (open); Heyer and Rose |
| Component B threshold | B = fixed 16 wk (not data-derived) | 3 | 2 / 1 / 0 | 1 | 0.82 | Nikolopoulos et al.; López Valerio et al.; Heyer and Rose |
| Component B threshold | B = technique-specific (E 11.80; O 16.89 wk) | 5 | 4 / 1 / 0 | 3 | 0.55 | Nikolopoulos et al.; Sugimoto et al.; Smyth et al.; López Valerio et al.; Heyer and Rose |
| Component B threshold | B = best available milestone (13.30 wk) | 5 | 3 / 2 / 0 | 1 | 0.85 | Nikolopoulos et al.; Sugimoto et al.; López Valerio et al.; Ballal et al. (open); Heyer and Rose |
| Missing data | B missing = 1 (worst case for arms without a return time) | 7 | 3 / 3 / 1 | 3 | 0.61 | Nikolopoulos et al.; López Valerio et al.; Ballal et al. (open); Ahn et al. (endoscopic); Ahn et al. (subtalar arthroscopy); Walsh et al.; Heyer and Rose |
| Any-event components | A and D rate-based (> mean + 1 SD of arm rates: A 3.6%, D 12.8%) | 2 | 0 / 2 / 0 | 2 | 0.60 | Ballal et al. (open); Heyer and Rose |
| Component C (MCID) | C = highest eligible MCIDs (AOFAS 30.2; VAS 5.2) | 10 | 5 / 4 / 1 | 6 | 0.35 | Nikolopoulos et al.; Sugimoto et al.; López Valerio et al.; Georgiannos and Bisbinas (endoscopic); Georgiannos and Bisbinas (open); Ballal et al. (open); Ahn et al. (endoscopic); Ahn et al. (subtalar arthroscopy); Walsh et al.; Heyer and Rose |

E, endoscopic; O, open; S, subtalar arthroscopy; κ, Cohen’s kappa against the primary classification. Only one element of the definition is varied in each row; all other elements are as in the primary specification (≥ 2 of 4 components; component B at the mean plus 1 SD of the 11 arms reporting a full or prior-level return; component C at the primary MCIDs; any-event components A and D). Fixed cut-offs of 12 and 16 weeks are illustrative and not externally validated. Rate-based components score 1 when the arm’s rate exceeds the mean plus 1 SD of all arm rates (failed return: 3.6%; any complication: 12.8%). Missing data: arms without a full or prior-level return time scored 1 on component B (worst case). Highest eligible MCIDs: AOFAS change below 30.2 points or VAS change below 5.2 points. Arms score positive on component B only if their mean exceeds the unrounded threshold: the 90th percentile (14.90 weeks) equals the mean of the open arm of Ballal et al. (14.9 weeks), which is therefore not positive under that specification.

**Part B. Primary component scores, the events behind them, and stability of each arm across the 16 specifications (primary and 15 alternatives)**

| **Study arm** | **Technique** | **n** | **A** | **B (full return, wk)** | **C** | **D** | **Score** | **Primary classification** | **Classified in (of 16)** | **Events behind positive components** |
| --- | --- | --- | --- | --- | --- | --- | --- | --- | --- | --- |
| Nikolopoulos et al. | Endoscopic | 81 | 1 | 0 (–) | 0 | 1 | 2 | Poor-outcome profile | 14 | A: 5/81 returned at a lower activity level. D: 3 transient sural nerve neurapraxias. |
| Shimozono et al. | Endoscopic | 13 | 0 | 0 (6.3) | 0 | 0 | 0 | – | 0 | None. |
| Sugimoto et al. | Endoscopic | 50 | 0 | 0 (13.6) | 0 | 1 | 1 | – | 7 | D: 3 skin burns from monopolar cautery; 1 scar excision (reoperation). |
| Smyth et al. | Endoscopic | 16 | 0 | 0 (11.9) | 0 | 1 | 1 | – | 2 | D: 1 deep peroneal nerve dysaesthesia; 1 infection requiring debridement and washout. |
| López Valerio et al. | Endoscopic | 20 | 1 | 0 (6.7) | 0 | 1 | 2 | Poor-outcome profile | 14 | A: 1/20 did not regain the prior Tegner level. D: 1 reoperation for persistent pain. |
| Jerosch and Fadel | Endoscopic | 10 | 0 | 0 (–) | 0 | 0 | 0 | – | 0 | None. |
| Morelli et al. | Endoscopic | 12 | 0 | 0 (–) | 0 | 0 | 0 | – | 0 | None (local swelling in 1 patient does not meet component D). |
| Georgiannos and Bisbinas (endoscopic) | Endoscopic | 26 | 0 | 0 (7.12) | 0 | 1 | 1 | – | 2 | D: 1 revision endoscopy for persistent pain. |
| Georgiannos and Bisbinas (open) | Open | 26 | 0 | 0 (11.54) | 0 | 1 | 1 | – | 2 | D: 3 sural nerve injuries (1 permanent); 2 wound infections treated with antibiotics; 1 CRPS. |
| Ballal et al. (endoscopic) | Endoscopic | 13 | 0 | 0 (9.8) | 0 | 0 | 0 | – | 0 | None. |
| Ballal et al. (open) | Open | 22 | 1 | 1 (14.9) | 0 | 0 | 2 | Poor-outcome profile | 9 | A: 1/22 did not return to full ballet (unrelated hip injury). B: 14.9 > 14.55 weeks. |
| Guo et al. (endoscopic) | Endoscopic | 25 | 0 | 0 (6.0) | 0 | 1 | 1 | – | 1 | D: 1 tibial nerve sensory loss (medial heel numbness). |
| Guo et al. (open) | Open | 16 | 0 | 0 (11.9) | 0 | 1 | 1 | – | 1 | D: 1 permanent sural nerve sensory loss. |
| Ahn et al. (endoscopic) | Endoscopic | 12 | 0 | 0 (–) | 0 | 1 | 1 | – | 3 | D: 1 transient sural nerve dysaesthesia. |
| Ahn et al. (subtalar arthroscopy) | Subtalar arthroscopy | 16 | 0 | 0 (–) | 0 | 1 | 1 | – | 3 | D: 1 transient sural nerve dysaesthesia. |
| Walsh et al. | Open | 44 | 0 | 0 (–) | 0 | 1 | 1 | – | 3 | D: 2 temporary sural nerve injuries. |
| Heyer and Rose | Open | 38 | 1 | 1 (17.7) | 0 | 1 | 3 | Poor-outcome profile | 16 | A: 2/37 did not return to prior dance level. B: 17.7 > 14.55 weeks. D: 1 superficial infection and 1 wound dehiscence. |

– in column B: no full or prior-level return time reported, so component B was not assessable and scored 0. Arms classified identically in every specification: 5 of 17 (Heyer and Rose always; Shimozono et al., Jerosch and Fadel, Morelli et al. and the endoscopic arm of Ballal et al. never). Excluding the ≥ 1 and ≥ 3 rules, 7 of 17 arms were classified identically in all 14 remaining specifications. CRPS, complex regional pain syndrome.

**Part C. Leave-one-study-out derivation of the component B threshold**

| **Study omitted** | **Arms contributing to threshold** | **Threshold (weeks)** | **Arms changing on B** | **Arms changing classification** | **Arm(s) affected** |
| --- | --- | --- | --- | --- | --- |
| Nikolopoulos et al. | 11 | 14.55 | 0 | 0 | – |
| Shimozono et al. | 10 | 14.90 | 0 | 0 | – |
| Sugimoto et al. | 10 | 14.33 | 0 | 0 | – |
| Smyth et al. | 10 | 14.61 | 0 | 0 | – |
| López Valerio et al. | 10 | 14.91 | 1 | 1 | Ballal et al. (open) |
| Jerosch and Fadel | 11 | 14.55 | 0 | 0 | – |
| Morelli et al. | 11 | 14.55 | 0 | 0 | – |
| Georgiannos and Bisbinas (both arms) | 9 | 15.09 | 1 | 1 | Ballal et al. (open) |
| Ballal et al. (both arms) | 9 | 14.34 | 0 | 0 | – |
| Guo et al. (both arms) | 9 | 15.02 | 1 | 1 | Ballal et al. (open) |
| Ahn et al. (both arms) | 11 | 14.55 | 0 | 0 | – |
| Walsh et al. | 11 | 14.55 | 0 | 0 | – |
| Heyer and Rose | 10 | 13.23 | 1 | 1 | Sugimoto et al. |

Every arm, including those of the omitted study, was scored against the threshold derived without that study. Omitting a study without a full or prior-level return time leaves the threshold at 14.55 weeks. Range of thresholds: 13.23 to 15.09 weeks. Thresholds are rounded to two decimal places; omitting Shimozono et al. gives 14.896 weeks, so the open arm of Ballal et al. (14.9 weeks) remains positive on component B.

**Part D. Arm size and the any-event components**

| **Study arm** | **n** | **Failed return n/N (%)** | **A any-event** | **A rate-based** | **Any complication n/N (%)** | **D any-event** | **D rate-based** | **Primary score** | **Rate-based score** |
| --- | --- | --- | --- | --- | --- | --- | --- | --- | --- |
| Nikolopoulos et al. | 81 | 5/81 (6.2) | 1 | 1 | 5/81 (6.2) | 1 | 0 | 2 | 1 |
| Shimozono et al. | 13 | 0/13 (0) | 0 | 0 | 0/13 (0) | 0 | 0 | 0 | 0 |
| Sugimoto et al. | 50 | 0/49 (0) | 0 | 0 | 4/72 (5.6) | 1 | 0 | 1 | 0 |
| Smyth et al. | 16 | 0/16 (0) | 0 | 0 | 2/22 (9.1) | 1 | 0 | 1 | 0 |
| López Valerio et al. | 20 | 1/20 (5) | 1 | 1 | 1/20 (5) | 1 | 0 | 2 | 1 |
| Jerosch and Fadel | 10 | 0/10 (0) | 0 | 0 | 0/10 (0) | 0 | 0 | 0 | 0 |
| Morelli et al. | 12 | 0/12 (0) | 0 | 0 | 1/12 (8.3) | 0 | 0 | 0 | 0 |
| Georgiannos and Bisbinas (endoscopic) | 26 | 0/26 (0) | 0 | 0 | 1/26 (3.8) | 1 | 0 | 1 | 0 |
| Georgiannos and Bisbinas (open) | 26 | 0/26 (0) | 0 | 0 | 6/26 (23.1) | 1 | 1 | 1 | 1 |
| Ballal et al. (endoscopic) | 13 | 0/13 (0) | 0 | 0 | 0/13 (0) | 0 | 0 | 0 | 0 |
| Ballal et al. (open) | 22 | 1/22 (4.5) | 1 | 1 | 0/22 (0) | 0 | 0 | 2 | 2 |
| Guo et al. (endoscopic) | 25 | 0/25 (0) | 0 | 0 | 1/25 (4) | 1 | 0 | 1 | 0 |
| Guo et al. (open) | 16 | 0/16 (0) | 0 | 0 | 2/16 (12.5) | 1 | 0 | 1 | 0 |
| Ahn et al. (endoscopic) | 12 | 0/12 (0) | 0 | 0 | 1/12 (8.3) | 1 | 0 | 1 | 0 |
| Ahn et al. (subtalar arthroscopy) | 16 | 0/16 (0) | 0 | 0 | 1/16 (6.3) | 1 | 0 | 1 | 0 |
| Walsh et al. | 44 | 0/44 (0) | 0 | 0 | 6/44 (13.6) | 1 | 1 | 1 | 1 |
| Heyer and Rose | 38 | 2/37 (5.4) | 1 | 1 | 4/40 (10) | 1 | 0 | 3 | 2 |

Component D any-event uses the pre-specified definition (nerve complication, CRPS, wound complication requiring active management, or reoperation); the rate-based version uses the any-complication rate reported for each arm. Spearman correlation of arm size with composite score: ρ 0.66 (p = 0.004) for the primary specification and ρ 0.56 (p = 0.018) for the rate-based specification. Median arm size: 30 in classified arms and 16 in unclassified arms (primary specification).

**Part E. Change scores expressed in MCID units (MCID normalisation)**

| **Study arm** | **AOFAS change** | **MCID 5.5** | **MCID 8 (primary)** | **MCID 30.2** | **VAS change** | **MCID 1.8** | **MCID 2.0 (primary)** | **MCID 5.2** |
| --- | --- | --- | --- | --- | --- | --- | --- | --- |
| Nikolopoulos et al. | 58.3 | 10.6 | 7.3 | 1.93 | 6.9 | 3.8 | 3.5 | 1.33 |
| Sugimoto et al. | 18.9 | 3.4 | 2.4 | 0.63 | – | – | – | – |
| López Valerio et al. | – | – | – | – | 6.7 | 3.7 | 3.4 | 1.29 |
| Jerosch and Fadel | 44.0 | 8.0 | 5.5 | 1.46 | – | – | – | – |
| Morelli et al. | 28.2 | 5.1 | 3.5 | 0.93 | 5.3 | 2.9 | 2.6 | 1.02 |
| Georgiannos and Bisbinas (endoscopic) | 26.6 | 4.8 | 3.3 | 0.88 | – | – | – | – |
| Georgiannos and Bisbinas (open) | 21.4 | 3.9 | 2.7 | 0.71 | – | – | – | – |
| Ahn et al. (endoscopic) | 25.1 | 4.6 | 3.1 | 0.83 | 5.5 | 3.1 | 2.8 | 1.06 |
| Ahn et al. (subtalar arthroscopy) | 26.1 | 4.7 | 3.3 | 0.86 | 5.1 | 2.8 | 2.5 | 0.98 |
| Walsh et al. | – | – | – | – | 3.66 | 2.0 | 1.8 | 0.70 |
| Heyer and Rose | – | – | – | – | 7.1 | 3.9 | 3.5 | 1.37 |

MCID units = change score ÷ MCID (Ramadanov [1]); 1.0 corresponds to one minimal clinically important improvement. MCID values are the lowest and highest eligible estimates from the sources used in the worked example (AOFAS: 5.5 points, anchor-based, after unstable ankle fracture [2]; 30.2 points after hallux valgus surgery [3]; VAS: 1.8 and 5.2 points after hallux valgus surgery [4]) and the primary values. Arms without a baseline AOFAS or VAS score cannot be normalised: Shimozono et al. and Smyth et al. used other instruments, and Ballal et al. and Guo et al. reported no baseline score. The SD of the change could not be derived because no study reported the pre–post correlation. VAS change is a reduction in pain.

**Part F. Direction of effect and confidence decision rules (Table 2) for each factor**

| **Factor** | **Stage 1: not determinable?** | **Starting level** | **Downgrades** | **Rating by rules** | **Original rating** | **Direction of effect** |
| --- | --- | --- | --- | --- | --- | --- |
| Technique: return to sport | No | High (randomised within the RCT) | As in the GRADE assessment (Supplementary Table S3) | Low | Low (GRADE) | Endoscopic faster: RCT 4.42 weeks (95% CI 2.69 to 6.15) |
| Technique: complications | No | High (randomised within the RCT) | As in the GRADE assessment (Supplementary Table S3); RCT OR 7.50, 95% CI 0.83 to 67.49, from 7 events | Very low | Very low (GRADE) | Fewer after endoscopic excision; imprecise |
| Sport or dancer status | No | Low (within-study contrasts in 4 studies: Shimozono et al., Guo et al., Smyth et al., Nikolopoulos et al.) | Inconsistency: none (3 of 4 contrasts, 75%, same direction; opposite in Smyth et al.); imprecision −1 (151 participants) | Very low | Very low | Dancers possibly slower within studies; not consistent across arms |
| FHL pathology | Yes: measured in non-comparable ways, no within-study contrast | – | – | Not determinable | Insufficient data | Not determinable |
| Symptom duration | No (8 arms) | Very low (between arms only) | – (already very low) | Very low | Very low | No pattern |
| Ossicle size and concomitant pathology | Size: yes (collinear with technique in the only study that measured it); concomitant pathology: no | Size: –; concomitant pathology: very low (between arms only) | Concomitant pathology: inconsistency (already very low) | Size: not determinable; concomitant pathology: very low | Very low (combined row) | Large ossicle: technical difficulty with subtalar arthroscopy (2/16 converted); concomitant pathology: no consistent pattern |
| Surgeon experience | No: collinear with technique in 2 studies but not in Sugimoto et al. | Low (within-study learning-curve analysis, 1 study) | Imprecision −1 (single study) | Very low (no effect on clinical outcome shown) | Not testable across arms | Operating time falls with experience; no effect on clinical outcome shown |
| Rehabilitation protocol | Yes: no variation (early mobilisation in every arm) | – | – | Not determinable | Not testable | No late-mobilisation comparator |

Ratings for technique follow the GRADE assessment of the randomised comparison (Supplementary Table S3), as Table 2 specifies. The rules reproduced the original rating for every row except surgeon experience, where both ratings indicate that the evidence cannot support a conclusion, and ossicle size, which the rules rate not determinable because size was collinear with technique in the only study that measured it (Ahn et al.). Two reviewers applied the rules independently.

**Part G. Moderator sensitivity analyses: direction of the technique contrast in restricted sets of arms**

| **Set of arms** | **Arms (E / O)** | **Full or prior-level return, unweighted mean of arm means, weeks: E vs O (arms)** | **Any complication, events/N: E vs O** | **Direction preserved** |
| --- | --- | --- | --- | --- |
| All arms (reference) | 11 / 5 | 8.77 (7) vs 14.01 (4) | 16/306 (5.2%) vs 18/148 (12.2%) | – |
| Excluding the six dancer-predominant arms (Shimozono et al., Morelli et al., Ballal et al. both arms, Walsh et al., Heyer and Rose) | 8 / 2 | 9.06 (5) vs 11.72 (2) | 15/268 (5.6%) vs 8/42 (19.0%) | Yes, both outcomes |
| Arms of the four studies that explicitly excluded concomitant pathology (Nikolopoulos et al., Morelli et al., Georgiannos and Bisbinas, Ahn et al.) | 4 / 1 | 7.12 (1) vs 11.54 (1); randomised comparison only | 8/131 (6.1%) vs 6/26 (23.1%) | Yes, both outcomes |

Descriptive comparisons of unweighted arm means and crude event proportions; the subtalar arthroscopy arm (Ahn et al.) is not part of the technique contrast. Complication denominators follow Supplementary Table S4 Part D. In the restricted set excluding concomitant pathology, only the randomised trial reports a full or prior-level return in both techniques. E, endoscopic; O, open.

**References**

[1] N. Ramadanov, MCID normalization: a methodological framework for harmonizing heterogeneous PROMs in hip arthroscopy research, J. Exp. Orthop. 12 (2025) e70568. https://doi.org/10.1002/jeo2.70568.

[2] J. Tausendfreund, D. Penning, E. Ritchie, B. Twigt, P. Joosse, S. van Dieren, T. Schepers, The minimal clinically important difference of the American Orthopaedic Foot and Ankle Society Ankle Hindfoot Scale in patients with unstable ankle fracture, Clin. Orthop. Surg. 18 (2026) 337–342. https://doi.org/10.4055/cios25241.

[3] H.Y. Chan, J.Y. Chen, S. Zainul-Abidin, et al., Minimal clinically important differences for American Orthopaedic Foot & Ankle Society Score in hallux valgus surgery, Foot Ankle Int. 38 (2017) 551–557.

[4] R.M. Sutton, E.L. McDonald, R.J. Shakked, D. Fuchs, S.M. Raikin, Determination of minimum clinically important difference (MCID) in visual analog scale (VAS) pain and foot and ankle ability measure (FAAM) scores after hallux valgus surgery, Foot Ankle Int. 40 (2019) 687–693.
